# COVID-19-Associated Fungal Co-Infections: Pathogenesis, Diagnostics, Biomarkers, and Therapeutic Strategies

**DOI:** 10.3390/jcm15176896

**Published:** 2026-09-06

**Authors:** Swapnila Choudhury, Nafisa Nawaar, Woasifur Rahman Chowdhury, Puja Roy, Auroni Semonti Khan, Sajad Ali, Randa Mohammed Zaki, Topu Raihan, Meerambika Mishra, Muhammad Fazle Rabbee, Kwang-Hyun Baek

**Affiliations:** 1 Department of Genetic Engineering and Biotechnology, Jagannath University, Dhaka 1100, Bangladesh; 2 Department of Biochemistry and Microbiology, North South University, Dhaka 1229, Bangladesh; 3 Department of Genetic Engineering and Biotechnology, Shahjalal University of Science and Technology, Sylhet 3114, Bangladesh; 4 Department of Biological Sciences, College of Science, King Faisal University, Al-Ahsa 31982, Saudi Arabia; 5 Department of Pharmaceutics, College of Pharmacy, Prince Sattam Bin Abdulaziz University, Al-Kharj 11942, Saudi Arabia; 6 Department of Pharmaceutics and Industrial Pharmacy, Faculty of Pharmacy, Beni-Suef University, Beni-Suef 62514, Egypt; 7 Faculty of Life and Environmental Science, Shimane University, Matsue 690-8504, Japan; 8 Department of Infectious Disease and Immunology, College of Veterinary Medicine, University of Florida, Gainesville, FL 32611, USA; 9 Department of Biotechnology, Yeungnam University, Gyeongsan 38541, Republic of Korea

**Keywords:** fungal co-infections, SARS-CoV-2 outbreaks, immune response, signal transduction, antifungal drugs

## Abstract

From a health perspective, the 21st century has witnessed the emergence and global impact of several major viral diseases, most notably coronavirus disease 2019 (COVID-19) caused by SARS-CoV-2, which has been associated with a substantial burden of secondary fungal infections. Patients suffering from COVID-19 illnesses frequently developed fungal co-infections, which can worsen clinical outcomes and complicate therapeutic efforts. Hospitalized individuals with viral infections are particularly susceptible to invasive fungal pathogens, including *Aspergillus*, *Candida*, and *Mucorales* species. The co-pathogenesis between respiratory virus and fungi is complex, involving dynamic interactions among the pathogens and the host immune system. Opportunistic fungal infections were found to be more prevalent in COVID-19-infected individuals, who require mechanical ventilation, have diabetes, or exhibit neutropenia. This review aims to provide a comprehensive overview of fungal co-infections associated with COVID-19 disease, with a focus on their pathogenesis, biomarkers, diagnostic approaches, and potential treatment strategies. Overall, the available evidence indicates that viral-induced immune dysregulation, epithelial barrier damage, and clinical risk factors contribute to the development and severity of fungal co-infections in COVID-19 patients. Early recognition using reliable biomarkers and standardized diagnostic approaches, together with timely and pathogen-directed antifungal therapy, are essential for improving clinical outcomes.

## 1. Introduction

In recent decades, infectious disease epidemics have been on the rise and have posed threats to the global economy, biodefense systems, and health security. Following the emergence of the novel coronavirus SARS-CoV-1 in China in 2002, the World Health Organization (WHO) established the WHO Emergency Committee in 2005 to enhance global preparedness and mitigate the risks posed by emerging infectious agents [1]. In recent decades, infectious disease epidemics have been on the rise and have posed threats to the global economy, biodefense systems, and health security. Following the emergence of the novel coronavirus SARS-CoV-1 in China in 2002, the World Health Organization (WHO) established the WHO Emergency Committee in 2005 to enhance global preparedness and mitigate the risks posed by emerging infectious agents [1]. Over the past three decades, a succession of newly discovered viral outbreaks has spread worldwide. Notable examples include the influenza A pandemic in early 2009 in Mexico, the Middle East Respiratory Syndrome (MERS) outbreak in 2012 in Saudi Arabia [2], yellow fever outbreak in Brazil, in 2016 [3], West African Ebola virus outbreak in late 2013, the polio outbreak in 2014, the Lassa virus-caused health emergence in Nigeria in 2018, and the most recent coronavirus disease 2019 (COVID-19) outbreak caused by severe acute respiratory syndrome coronavirus 2 (SARS-CoV-2) [4].

SARS-CoV-2, has emerged as a global public health crisis since its onset in 2019 [5]. According to WHO, this pandemic has affected over 779 million individuals worldwide with almost 7 million deaths till date [6]. One-third of hospitalized patients developed severe acute respiratory distress syndrome, which necessitated intensive care unit (ICU) admission and mechanical ventilation [7]. Respiratory virus infections like COVID-19 can become more severe when they coexist with other infectious fungi [8]. Fungal co-infections are detrimental to human health as they often intensify the severity of viral diseases [9,10]. Although the precise mechanisms underlying these interactions have not been clearly elucidated, two possible mechanisms have been suggested for the co-infecting pathogens: direct contact or indirect interaction mediated by the host immune system [11,12,13]. Fungal co-infections in patients with viral illnesses have received less attention prior to the COVID-19 outbreak. However, after COVID-19 patients experiencing multiple types of fungal co-infection, it became a major public health concern. The commonly found fungal pathogens as infectious agents are from the fungal genera *Aspergillus*, *Cryptococcus*, *Candida*, *Mucor*, *Saccharomyces*, and *Pneumocystis* and they contribute to mortality invasive fungal infections associated with SARS-CoV-2 viral disease. Invasive pulmonary aspergillosis (IPA), COVID-19-associated pulmonary aspergillosis (CAP), COVID-19-associated mucormycosis (CAM), Cryptococcosis, etc., can easily develop in patients infected with SARS-CoV-2 as well as in other viral diseases.

The co-infection with fungi in COVID-19 patients poses severe challenges in treatment of critically ill patients. Fungal co-infections can further worsen clinical outcomes by increasing disease severity, prolonging hospitalization, and contributing to higher mortality [14]. However, existing knowledge on the pathomechanism involved in the invasion and pathogenesis is limited. Though fungal co-infections with viral illnesses are prevalent and have huge socioeconomic effects; studies on this aspect have lagged behind those of other pathogenic invasions. Furthermore, the lack of a standardized co-infection diagnosis underscores the need for another type of research aimed at assessing the relationships between the risk factors for fungal co-infections, available therapies, and co-fatalities. This review paper aimed to investigate the prevalence of fungal co-infections during the COVID-19 pandemic. Moreover, the processes of infection, challenges in diagnosing of fungal pathogens, and treatment concerns unique to fungal infections associated with COVID-19 are included in this study.

## 2. Literature Search Strategies

A literature search was conducted to identify relevant studies on fungal co-infections associated with COVID-19. The search was performed using PubMed, Web of Science, Medline, Scopus, and Google Scholar. Relevant publications were identified using combinations of the following keywords and Boolean operators: “COVID-19” OR “SARS-CoV-2” OR AND “fungal infection” OR “fungal co-infection” OR “secondary fungal infection” OR “invasive fungal infection” OR “aspergillosis” OR “mucormycosis” OR “candidiasis” AND “pathogenesis” OR “diagnosis” OR “biomarkers” OR “treatment” OR “therapeutic strategies.” Articles were selected based on their relevance to the objectives and scope of the review, with particular emphasis on studies addressing the epidemiology, pathogenesis, clinical manifestations, diagnostic approaches, biomarkers, and therapeutic strategies for fungal infections associated with COVID-19 and Influenza.

## 3. Fungal Co-Infection During COVID-19 Pandemic

**Figure 1:**
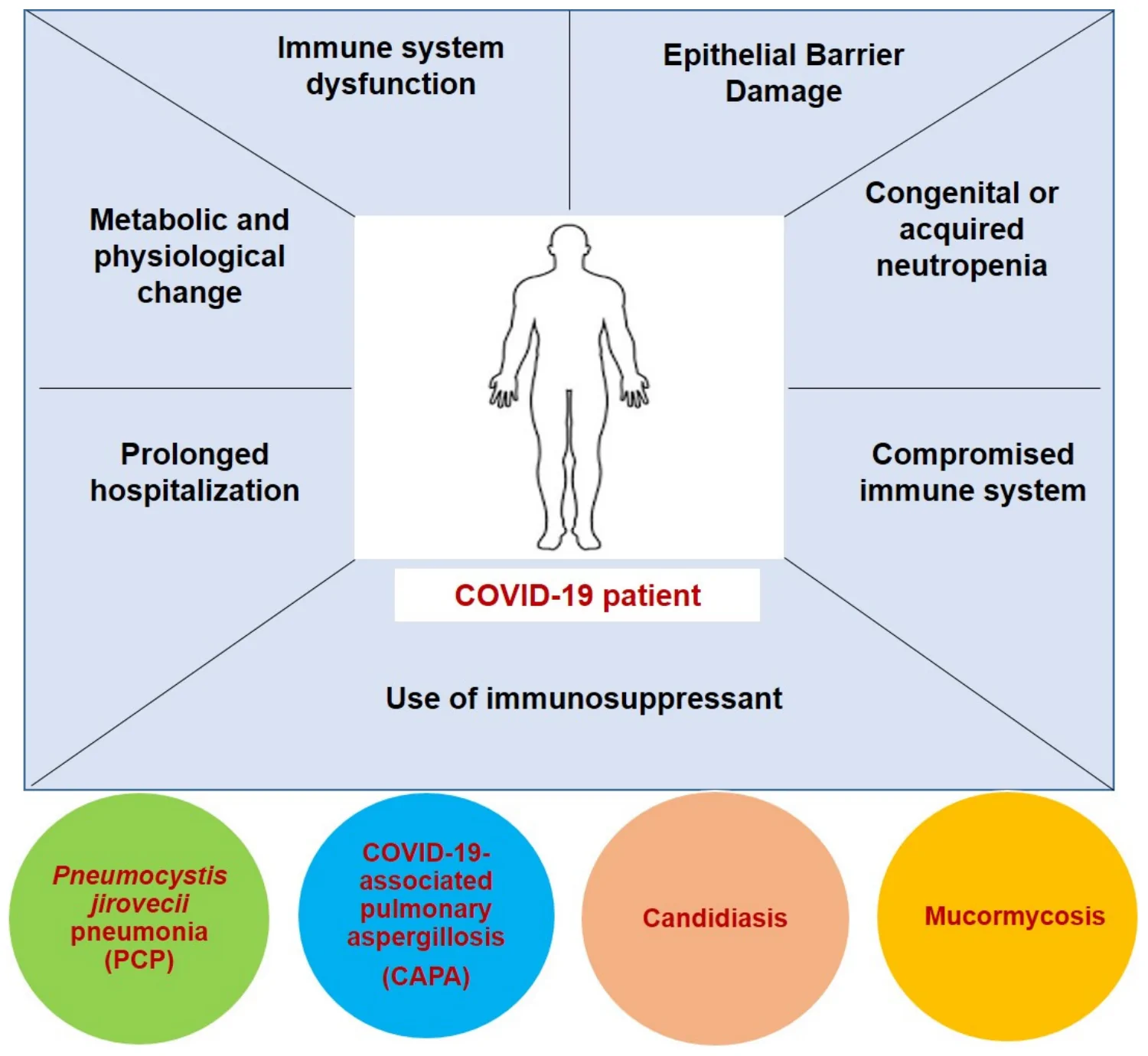
Risk factors predisposing COVID-19 patients to opportunistic fungal infections. COVID-19-associated conditions, such as immune system dysfunction, epithelial barrier damage, congenital or acquired neutropenia, metabolic and physiological changes, prolonged hospitalization, compromised immunity, and the use of immunosuppressants, collectively increase susceptibility to secondary fungal diseases, such as *Pneumocystis jirovecii pneumonia* (PCP), COVID-19-associated pulmonary aspergillosis (CAPA), candidiasis, and mucormycosis.

Patients with COVID-19 were extremely susceptible to various fungal pathogens, including *Aspergillus flavus*, *Candida albicans*, *C. glabrata*, and Mucorales [15,16]. The risk of death in patients with COVID-19 had more than doubled due to bacterial and fungal co-infections [17]. Fungal pathogenesis in patients with COVID-19 include aspergillosis, mucormycosis (fungal infection caused by molds belonging to the order Mucorales), candidiasis, and other lethal fungal infections, resulting in high morbidity and mortality rates [16,18]. Fungal co-infection in patients infected by SARS-CoV-2 has been extensively investigated owing to the frequent occurrence of fungal co-infections (Figure 1).

For example: (1). COVID-19-associated candidiasis, caused by *Candida* spp. (was initially reported in 2019 following the emergence of SARS-CoV-2 [18]. *C. albicans* remains the most frequently reported etiological agent of candidiasis, accounting for approximately 75% of cases; however, accumulating evidence from the COVID-19 era underscores the increasing clinical relevance of non-albicans *Candida* species (NAC), including *Meyerozyma parapsilosis*, *Meyerozyma guilliermondii*, *Nakaseomyces glabratus*, *Clavispora lusitaniae*, *Candida tropicalis*, *Candida krusei*, and *Candida dubliniensis*. The key virulence factor for candidiasis includes ability of the pathogens to adhere to epithelial and endothelial surfaces through adhesins. Following adhesion, the pathogen is either uptake by the host cell through endocytosis or by fungi driven penetration into the host cell by breaking barrier. It is followed by the yeast-hyphae transition and formation of protective biofilm that aids in escaping immune surveillance and development of infection [19]. Critically ill patients with COVID-19—particularly those receiving broad-spectrum antibiotics or corticosteroids, parenteral nutrition, or experiencing prolonged intensive care unit stays—exhibit heightened susceptibility to invasive infections caused by NAC [20,21]. Collectively, the rising prevalence of NAC in COVID-19-associated candidemia highlights the need for species-level identification, targeted antifungal therapy, and strengthened hospital infection-control practices.

(2). COVID-19-associated pulmonary aspergillosis (CAPA), initially reported in early 2020 in China, is an additional complication of COVID-19 treatment due to the invasive growth of *Aspergillus* spp. (predominantly *A. fumigatus*) in the airways [22]. Aspergillosis is initiated once the inhaled *Aspergillu* spore reaches to the alveoli of the lungs and the conidia starts to germinate. The invasive *Aspergillu* hyphae could penetrate the lung tissue resulting in the tissue damage and diseases progression [23]. The lung injury in the COVID-19 patients due to the viral attack often worsens the situation because the impaired immune system along with epithelial barrier dysfunction and impaired phagocytic activity against fungi results in increased susceptibility to CAPA [24].

(3). COVID-19-associated mucormycosis (CAM), a rare fungal infection caused by a group of molds known as mucormycetes, gained worldwide attention in 2021 during the second wave of the COVID-19 pandemic in India, with a prevalence of 70 times higher than in the rest of the world [25,26]. *Rhizopus arrhizus* was the predominant fungal pathogen in CAM in India [16]. Fungal spores can enter patients through various channels, such as inhalation, catheters, and surgical equipment, infecting the compromised immune systems of patients with COVID-19, which are already weakened by the use of medications (e.g., steroids) that dampen the viral effects [27,28]. The spores are endocytosed and germinates to give rise to hyphae—the reason for ultimate endothelial damage and hematogenous dissemination. The interaction between the fungus and endothelial cells around blood vessels is an important step in mucormycosis pathogenesis [29]. Mucormycosis can also evade crucial protective mechanisms of the host innate immune system, such as phagocytosis by macrophages and neutrophils, and other antiviral mechanisms of the immune system [30].

(4). The Cryptococcus species like *C. neoformans* and *C. gattii* are also prominent agents for fungal co-infection. The capsule of the yeast serves as a key virulence factor during infection inside the lungs. With the aid of capsule and other strategies, *Cryptococcus* successfully evade the immune attack and successfully establish the infections [31].

(5). *Pneumocystis jirovecii* pneumonia (PCP) is a life-threatening fungal infection caused by opportunistic fungal pathogen *P. jirovecii* and mostly occur in immunocompromised COVID-19 patients. PCP and SARS-CoV2 are comparable in their treatment, clinical presentation, and impact on the respiratory system [32]. Previous studies have revealed a notable association between fungal co-infections and viruses, such as SARS-CoV-2 and H1N1, each exhibiting distinct mechanisms of action [11]. *Pneumocystis* exists in three morphologically distinct trophic forms namely trophozoites, sporozoites and cysts. After transmitting through airborne route, the trophozoites attaches to the alveolar cells of the lungs where the trophozoites either form cyst or the haploid trophozoite undergoes sexual conjugation to form diploid sporozoites. Diploid sporozites ultimately give rise to mature sporozoites followed by formation of thick-walled cysts that trigger inflammation and resultant *Pneumocystis* pneumonia [33].

The susceptibility to additional fungal infections is dependent upon the recognition of the virus by the host immune system, as it provides the virus with the opportunity to establish an infection, compromising the host immune system. Consequently, fungal pathogens can evade host defense mechanisms and infiltrate cells and tissues [11]. The degree or intensity of fungal co-infection depends on various host factors, such as the route of infection, host innate immunity (composed of physical barriers and effector cells, such as macrophages, neutrophils, and natural killer (NK) cells), adaptive immune response (such as B and T cells), and fungal virulence properties, making the disease more severe and difficult to treat [16].

## 4. Pathophysiological Mechanisms Predisposing COVID-19 Patients to Fungal Co-Infections

**Figure 2:**
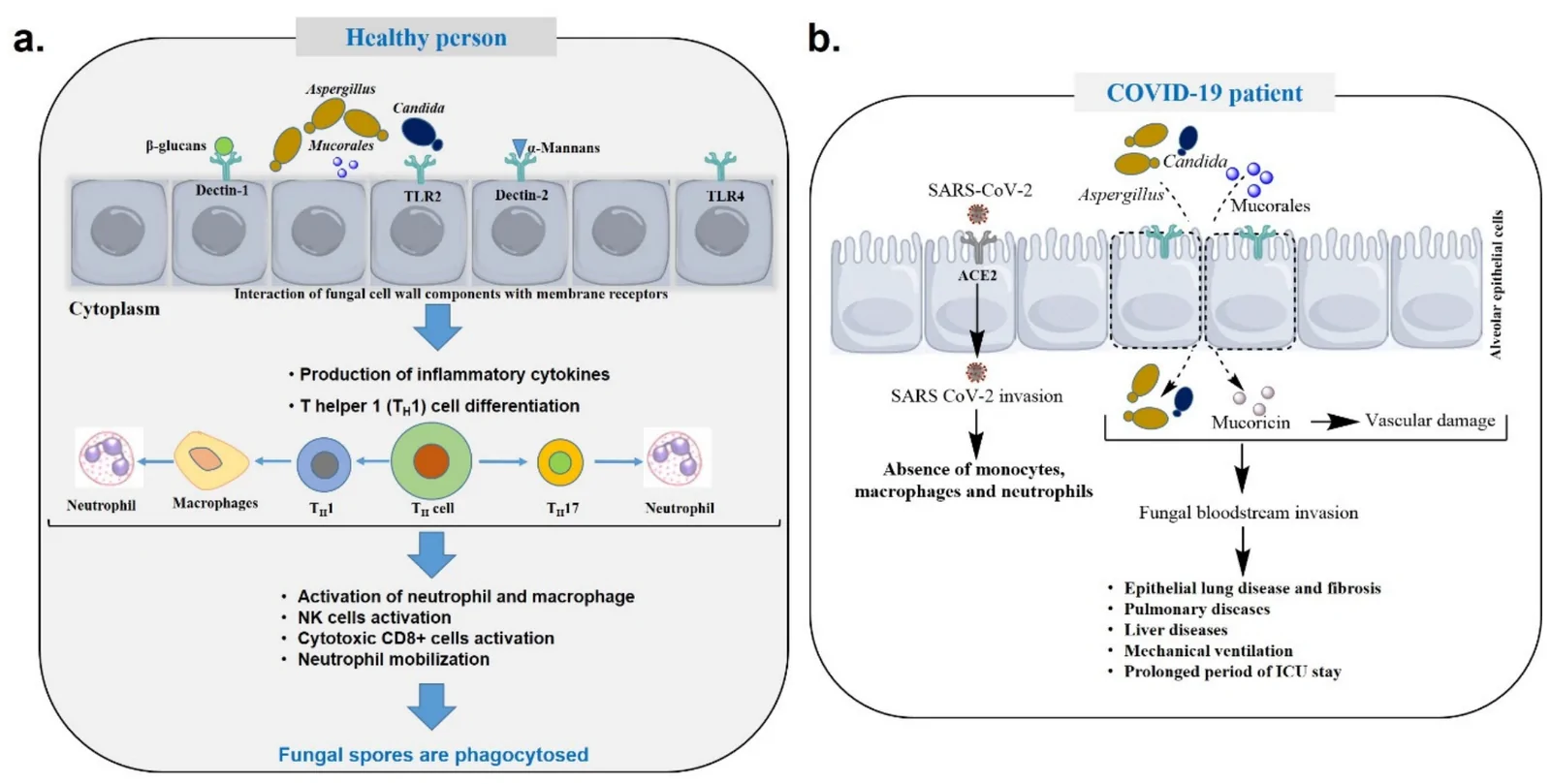
Complete pathophysiological cascade and clinical management trajectory of COVID-19-associated fungal co-infections. (**a**) In healthy individuals, fungal cell wall components, such as β-glucans, α-mannans, and other ligands from *Aspergillus*, *Candida*, and Mucorales bind host pattern recognition receptors (Dectin-1, TLR2, TLR4), triggering pro-inflammatory cytokine production and activating T_H_1, T_H_17 immune responses. Subsequent recruitment of activated neutrophils, macrophages, and cytotoxic CD8^+^ T cells, and NK cells achieves efficient fungal spore phagocytosis and tissue clearance. (**b**) Sequential pathogenesis, diagnosis, and management in COVID-19 patients. Stage 1: Viral entry and epithelial damage: SARS-CoV-2 engages ACE2 receptors on alveolar epithelial cells, initiating intracellular viral replication, cytopathic cell lysis, and disruption of the pulmonary epithelial barrier. Stage 2: Immune dysfunction: Systemic hyperinflammation combined with severe lymphopenia and iatrogenic immunosuppression depletes circulating and resident monocytes, macrophages, and neutrophils, disabling innate immune clearance. Stage 3: Fungal invasion and vascular pathology: Opportunistic germination of *Aspergillus*, *Candida*, and *Mucorales* leads to invasive hyphal growth, exotoxin release (e.g., mucoricin-mediated endothelial damage), angioinvasion, thrombosis, and tissue necrosis. Stage 4: Diagnostic identification: Multi-modal diagnostic confirmation via chest computed tomography. Stage 5: Clinical manifestations and targeted therapy: Progression to acute respiratory distress, pulmonary fibrosis, and multi-organ impairment requiring extended ICU stay and mechanical ventilation.

The epithelial barrier of the respiratory, gastrointestinal, and cutaneous surfaces serves as the first line of defense against fungal invasion. The envelope protein (E protein) of the SARS-CoV-2 acts as a virulence factor that signals to down-regulate the expression of tight junctional proteins that leads to the disruption of the airway epithelial barrier [34]. Disruption of these barriers significantly influences the pathophysiology of fungal infections, particularly in virus-infected hosts and increases susceptibility to opportunistic pathogens, including *A. fumigatus* and *C. albicans*. These fungi readily exploit compromised epithelial integrity to establish infection and promote disease progression [35]. When fungal entry occurs via the human respiratory tract, protection is provided by the respiratory epithelium, which contains ciliated cells along with a secretory cell lining that produces mucus [36]. In healthy individuals, inhaled fungal conidia are trapped within the mucus layer and mechanically removed by coordinated ciliary movement in the upper respiratory tract. However, COVID-19 patients are often prone to infection in the epithelial cells of the respiratory tract by SARS-CoV-2, compromising epithelial barrier integrity. Therefore, conidia continue to germinate and invade the epithelial tissue, ultimately resulting in fungal co-infection (Figure 2) [36].

Viral infection is initiated by the attachment of viral particles to specific host receptors [37]. The SARS-CoV-2 binds to the angiotensin-converting enzyme-2 receptor for host cell entry [38]. This receptor mediated attachment facilitates viral replication, transmission, and infection of cells in the upper and lower respiratory tracts, often leading to severe lung infections [39]. Invasion by respiratory viruses causes severe damage to the respiratory tract, including tracheal epithelial disruption, lung tissue damage, the alveolar epithelium inflammation, and epithelial tissue fibrosis [40,41]. Epithelial damage induced by viral infection creates favorable adhesion sites for fungal pathogens, such as *Aspergillus*, leading to the development of secondary infections in the respiratory tract of the human host. For example, *A. fumigatus* can bind to the α5β1 integrin apical receptor, which is upregulated in injured epithelial cells [42]. On human mucosal surfaces, *C. albicans* typically exists as a benign commensal organism; however, often causes infections under specific predisposing circumstances. The peptide toxin candidalysin, encoded by *ECE1* gene, damages host epithelial cells, and enhances fungal zinc uptake, a process critical for achieving maximal virulence during infection [43]. Additionally, *Candida* spp. recognizes and binds various forms of Toll-like receptors (TLRs), such as TLR2 and TLR4 [44].

Both innate and adaptive immunity of the human host are profoundly compromised during respiratory viral infections, thereby facilitating secondary fungal infections. Patients with COVID-19 experience significant T cell and NK cell response dysregulation, likely contributing to their increased susceptibility to fungal co-infections [45]. The suppression of T lymphocyte activity stimulates mucormycosis invasion in SARS-CoV-2-infected patients [46]. Also, dysregulated T helper cell type 17 (Th17) response during COVID-19 infection contribute to impaired inflammatory response as the production of IFN-17, a key component involved in antifungal immunity is disrupted. SARS-CoV-2 further causes the dysregulation of the adaptive immune response in the host. These eventually aids in the development of fungal co-infection [47]. In addition, the excessive activation of neutrophils and phagocytes can also have adverse effects, as unrestrained phagocyte activation ultimately damages the lungs and increases the risk of secondary fungal co-infection. In vivo mouse model experiments have demonstrated that the lungs were damaged due to excessive production of phagocytes, since neutrophil and macrophage activation increased the susceptibility to *C. gattii* co-infection and increased the fungal presence in the brain in influenza virus-infected patients [48].

## 5. Potential Risk Factors Associated with Fungal Co-Infections

### 5.1. Patients with Compromised Immune System

Individuals with compromised immune systems or other related conditions, such as diabetes, recent surgery, and prolonged hospital stays, were considered vulnerable to invasive fungal infections [49]. Many respiratory viruses, such as H1N1, SARS-CoV-2, SARS, and MERS disrupt the lung epithelium, increasing the risk of secondary fungal infection in damaged tissues. The lungs of severely infected patients with COVID-19 releases a host-derived factor called damaged-associated molecular patterns (DAMPs), which are associated with inflammation and lungs injury [50,51]. The secretion of DAMPs stimulates pathogen recognition by activating relevant receptors and regulating the host response to injury, resulting in a high incidence of CAPA [52]. CAPA is an excellent independent predictor of ICU mortality and is more prevalent in older patients, patients on invasive ventilation, and recipients of tocilizumab [53].

### 5.2. Prolonged Hospitalization

A common feature of patients with fungal co-infections is prolonged hospitalization, especially in those admitted to the ICU [54]. Prolonged hospitalization increases the risk of invasive fungal infections due to the utilization of ventilators and catheters. Mechanical ventilation often leads to fungal as well as bacterial co-infection as it increases the rate of microaspiration of contaminated oropharyngeal secretions [55]. For instance, a study of 197 COVID-19 patients treated in ICUs and on ventilators found that 68% of them had a variety of fungal co-infections, including *Candida* (75.4%), *Aspergillus* (16.4%), and *Mucor* (8.2%) [56]. Another study with 108 patients in Italy by Bartoletti et al. came up with a similar result, where a higher incidence of CAPA accounting for 27.7% were evident among COVID-19 patients placed to invasive mechanical ventilation [57]. This is because the apparatuses of mechanical ventilation provide fungal pathogens with access to the lungs, thereby increasing the risk of infection [12,51,58]. Previous studies examining *Candida* species isolated from patients with COVID-19 have confirmed that the risk factors associated with hospital environments are particularly linked to *Candida* co-infection [59,60].

### 5.3. Use of Immunosuppressant

A compromised immune system is a common risk factor for infections caused by *Aspergillus*, *Candida*, and *Rhizopus* spp., although *Aspergillus* infections are predominant in most cases [59]. Moreover, the use of immunosuppressants, such as methylprednisolone, dexamethasone, hydrocortisone, prednisone and tocilizumab, broad-spectrum antibiotics, and corticosteroids is associated with an increased risk of invasive fungal infections. Corticosteroids are frequently administered to reduce inflammation during viral and bacterial infections by downregulating the immune response. However, the immunosuppressive effects of these drugs render patients vulnerable to opportunistic fungal pathogens. The use of corticosteroids has been correlated with the exacerbation of patient’s condition and, consequently, a higher incidence of ICU admission [61,62]. Broad-spectrum antibiotics indirectly promote the colonization of fungal pathogens by disrupting the microflora. This eliminates competition and increases the risk of *Candida* and *Aspergillus* infection [59,63]. Furthermore, the emergence of multidrug-resistant strains complicates the situation, making the management of fungal co-infections even more challenging. The intensity of invasive fungal infections in virus-infected patients may also be influenced by associated conditions, such as uncontrolled diabetes mellitus, smoking habits, prior lung disease, or advanced age [60]. Even recent surgery and its associated impact may facilitate fungal invasion of human hosts. However, healthy individuals without a history of recent surgery, organ transplantation, or diabetes have also been reported to develop secondary fungal infections [64]. A study conducted among 135 COVID-19 patients in several ICUs in Wales reported that the use of high-dose corticosteroids significantly increased the occurrence of fungal co-infection to 26.7% aspergillosis and yeast infection [65]. Riche and co-workers in Brazil observed 10 times increase in candida co-infection among critically ill COVID-19 patients receiving high doses of corticosteroids [66]. Another study in Chicago involving 111 COVID-19 patients found out that receiving tocilizumab led to increased risk of developing fungal pneumonia and sinusitis [67]. Therefore, data from these studies indicate a strong relationship between immunosuppressants and fungal co-infection.

### 5.4. Congenital or Acquired Neutropenia

Congenital or acquired neutropenia (an inherited disorder with low number of neutrophils) is another important risk factor for invasive fungal co-infections. Neutropenia occurs in individuals as a result of hematologic malignancies, organ transplant, allogenic stem cell transplant [68], and cancer treated with chemotherapy or radiotherapy [69].

## 6. Prevalence of Fungal Infections During Viral Outbreaks in COVID-19

Fungal coinfections in patients with COVID-19 infections are highly heterogeneous, with reported prevalence varying according to disease severity, ICU exposure, diagnostic criteria, geographical setting, fungal species, and host risk factors. Across studies, invasive fungal infections were consistently more frequent among critically ill patients than among patients with mild or moderate illness. Many patients with COVID-19 are associated with severe fungal co-infection, which poses a serious health risk (Table 1). Furthermore, diagnosing fungal co-infection in COVID-19 patients is challenging, hampering the administration of appropriate treatment.

Among the fungal species involved in opportunistic fungal infections, the most prominent ones are *A. fumigatus*, *A. flavus*, *C. albicans*, and *C. glabrata* [95]. Opportunistic fungi, particularly *A. flavus*, *C. albicans*, and *C. glabrata*, infected 5% of patients with COVID-19 [95]. One study revealed that 27% of 48 patients with COVID-19 developed secondary fungal infections [15]. The fungal infection found to be more prevalent with higher rate of infection in patients admitted in ICU. For example, the prevalence rates of CAPA in SARS-CoV-2-infected ICU patients in France and Germany were 33.3% and 26.3%, respectively [71,96]. In a similar study conducted among 592 ICU patients with acute respiratory failure, 1.9% were diagnosed with proven CAPA, whereas 13.5% and 3.0% fulfilled probable and possible CAPA criteria, respectively [53]. Prevalence of IPA in ICU patients was also reported as approximately 33% of COVID-19 patients with in the ICU developed additional suspected invasive lung aspergillosis infections [97]. Candidiasis and Mucorales were also found to be prominent fungal infections in ICU admitted patients. One study reported that 61.53% of COVID-19 patients admitted in ICU were co-infected with *C. albicans* [98]. Severely ill and ICU admitted COVID-19 patients were also found to be more prone to mucormycosis and resultant comorbidity [99].

Geographic variation was particularly pronounced for COVID-19-associated secondary fungal infection. According to reports, there are up to 140 instances of CAM per million people in Sri Lanka, which is 80 times higher than that in developed countries [100]. In contrast to the higher reported burden in Sri Lanka, a study from Netherlands reported that 6 of 31 (19.4%) patients admitted to the ICU developed IPA in 2021 [101], with *A. fumigatus* being reported as the predominant fungal species causing IPA in critically ill patients [102]. In India during the second wave of COVID-19 in 2021, Mucorales (63.82%) were the most frequently isolated fungal infections, followed by *Aspergillus* spp. (14.89%) and *Candida* spp. (12.76%) in tertiary care institutions [103]. The overall mortality rate from invasive fungal infections in COVID-19 patients ranged from 40.25% to 71.4%, which was alarmingly high [104]. Collectively, these findings indicate substantial geographic heterogeneity in the incidence of COVID-19-associated fungal infections.

The occurrence of different fungal species in COVID-19 patients also varies. Aspergillosis is the most prevalent fungal coinfection followed by Candidiasis, Mucormycosis and others [105]. The reported incidence and prevalence of CAPA vary considerably across studies. This variation may be attributed to differences in patient risk factors, local epidemiological patterns, surveillance practices, diagnostic approaches, and the criteria applied for CAPA diagnosis. The mortality rate of CAPA infection is estimated to be approximately 40%; however, once CAPA becomes angioinvasive or invades the blood vessel walls, the mortality rate exceeds 80% [18]. The second most reported case of fungal coinfection is by *Candida*, which was classified as an “urgent threat” by the Centers for Disease Control and Prevention (CDC) due to its ability to cause systemic infections or bloodstream infections in critically ill patients [11]. Among *Candida* spp., *C. auris* is difficult to identify and is resistant to almost all available antifungal drugs [18]. COVID-19-associated *C. auris* infections result in a mortality rate of 30–83% in patients with candidemia [18]. The incidence of CAM is overwhelmingly higher in India than in other countries, due to the country having the highest number of patients with diabetes worldwide, with diabetes being a significant risk factor for mucormycosis [106]. Despite the severity of the situation in India, cases of mucormycosis have been documented in the USA, UK, Australia, France, Brazil, and Mexico. In addition to *Candida* infections, *Rhizopus* spp. is also one of the most frequently isolated fungal species in COVID-19 patients [107].

Considering the associated risk factors, a longer duration of ICU stay was observed among patients with fungal coinfections, suggesting that prolonged ICU hospitalization may be associated with an increased risk of fungal infection [108]. In one study, patients with long term corticosteroid treatment were found to be more susceptible to CAPA, making the prolonged corticosteroid treatment as possible risk factor [109]. Other risk factors for CAPA includes impaired immune response, extensive inflammation, and poor mucociliary activity. An investigation on the higher frequency of Candidiasis revealed that prolonged stay at hospital along with mechanical ventilation, catheters, and surgical procedure can be considered as significant risk factors in this regard [109]. Diabetes mellitus, malnutrition, and high BMI have been found to pose increased risk of Mucormycosis [110]. In some cases where the COVID-19 patients underwent corticosteroid, immunosuppressant and antibiotic treatments were diagnosed with increased occurrence of all types of fungal infection [109].

The reported prevalence and clinical outcomes of fungal coinfections among COVID-19 patients vary considerably across studies, reflecting substantial heterogeneity and uncertainty. This variation may arise from differences in patient populations, disease severity, underlying comorbidities, geographic and environmental factors, ICU exposure, corticosteroid and immunomodulatory therapy, and the duration of hospitalization. In addition, differences in case definitions and diagnostic criteria for CAPA, CAM, and other fungal infections may result in inconsistent classification across studies [110].

## 7. Mechanisms of Host Immunity in Viral-Fungal Co-Infections

The host innate and adaptive immune systems have adopted many pathways to eliminate the viral load and associated fungal co-infection. The antiviral response of the host body is predominantly mediated by the binding of a TLR, such as TLR3 or TLR7. These receptors are expressed on innate immune cells, such as dendritic cells or macrophages, which recognize viral RNA and trigger a signaling cascade that leads to the production of interferons and proinflammatory cytokines, such as interferons, interleukins, and tumor necrosis factors [111,112]. In the case of fungal co-infection, the C-type lectin receptors on dendritic cells play a major role in the detection of fungal components, such as glycolipids, glycoproteins, and glucans, and initiate multiple innate and adaptive immune responses against fungal pathogens [113]. The host immune system follows different pathways to combat the pathogens depending on the type of fungal pathogen present.

Various methods for eliminating or preventing viral and fungal pathogens have been elucidated to date, most of which require the participation of cellular and chemical components of innate and adaptive immune responses. IFN-mediated immune protection elicits antiviral and antifungal responses in human host cells [114]. Interferon I and III stimulate the expression of genes that can interfere with different steps of viral replication and terminate the process [111,112].

Upon recognition by *Aspergillus*, monocytes are activated and produce interferons to drive the antifungal response, which includes the activation of neutrophils and the production of reactive oxygen species (ROS) [115,116]. Also, once the *Aspergillus* conidia reach the lower respiratory tract from upper respiratory tract, a series of mechanisms facilitates their uptake into phagolysosomes, where they are degraded by the existing degradative enzymes and acidic environment (Figure 2) [36]. Neutrophils, macrophages, and monocytes are key components of the immune system that effectively protect against various pathogens [113]. The lung epithelium produces mucin and other soluble factors that prevent fungal invasion. Many pathogens are removed from the airways by mucociliary clearance, a process in which conidia are entrapped in mucus by binding between conidial lectins and glycan moieties on gel-forming mucins [117]. In addition, the immune system produces certain biochemical components, such as surfactant proteins (SP-A and SP-D), ficolins, and antimicrobial peptides, which can kill fungi by opsonization and phagocytosis [118]. Pro-inflammatory mediators, mucociliary clearance, and phagocytosis prevent secondary *A. fumigatus* infection [117]. The chemokine-mediated activation of macrophages, neutrophils, and other effector cells kills a significant number of fungal pathogens [44]. Natural killer (NK) cells play a key role in the cell-mediated killing of fungal pathogens, as NK cells directly kill fungi, along with its regulatory effects on the fungicidal activities of effector cells. A mouse model has shown that mice depleted of NK cells are more susceptible to infection by *A. fumigatus* [119]. In case of *C. albicans*, it is initially recognized by Toll-like receptors (TLRs), C-type lectin receptors (CLRs), NOD-like receptors (NLRs) and RIG-I-like receptors (RLRs) and triggers the adaptive immune response. Complement receptor 3 (CR3) and FcγRs are involved in the recognition of unopsonized and opsonized *Candida* by neutrophils, respectively. The effector cells like macrophage, neutrophil, NK cell along with helper T cell play key role in combatting candidiasis as well as mucorales [120,121].

## 8. Clinical Characteristics of Virus-Infected Patients with Fungal Co-Infection

The signs of invasive fungal infections manifest prominently in various tissues and organs of the host body, such as blood, lungs, and nasopharynx [122]. A radiography examination or computed tomography (CT) scan can be used to diagnose a fungal infection that first appears on the chest in patients infected with respiratory viruses. Thus, further confirmation through histology, blood culture, and sputum or bronchoalveolar lavage or BAL, may be required [95]. In some patients, whitish plaques were observed in the bronchi [123].

In cases of mucormycosis, black necrotic lesions appear on the palate, nasopharynx, sinuses, and orbit [124]. To establish a standard clinical diagnosis and classification of invasive fungal infections, the European Organization for Research and Treatment of Cancer and the Mycoses Study Group Education and Research Consortium published updated definitions of proven, probable, and possible fungal infections in 2019 [125]. According to the consensus, the microscopic detection of fungal hyphae in lung tissues or other tissues obtained by biopsy or needle aspiration is an indicator of invasive fungal infection by molds, such as *Aspergillus* and *Mucormycetes*. The presence of individual *Candida* cells in these tissue samples indicated invasive *Candida* infection. Probable invasive fungal infections are diagnosed by examining the sputum samples, BAL samples, or aspirates [126]. In most patients, fungal co-infections have been detected recently, which complicate the patient’s condition.

## 9. Fungal Biofilm That Intensify Viral–Fungal Co-Pathogenesis

The increasing use of medical implants, immunosuppressive therapies, and an ageing population has led to a rise in fungal biofilm-associated diseases, which are now recognized by the WHO as a significant threat to human health. These biofilms—structured communities of fungal cells attached to surfaces or enclosed within body cavities—are difficult to detect, remove, and treat due to their inherent tolerance to antifungal drugs. As a result, they contribute to serious clinical outcomes, including increased morbidity and mortality, repeated surgical interventions, and prolonged hospitalization [127,128,129]. For example, *Candida* biofilms resist phagocytosis by macrophages and inhibit the formation of neutrophil extracellular traps. In contrast, *Aspergillus* biofilms produce a unique extracellular polymeric matrix (ECM) that acts as a formidable shield, rendering biofilm-embedded cells largely invisible and impenetrable to innate immune cells. Moreover, ECM functions as a physical barrier that limits antifungal drug penetration, promotes drug sequestration, masks fungal pathogen-associated molecular patterns, contains extracellular DNA/RNA, secreted metabolites, and extracellular vehicles, which collectively impair phagocytosis, inhibit neutrophil extracellular trap formation, and suppress host immune responses [130]. Another study showed that *C. albicans* forms robust biofilms that confer protection against antifungal agents and host immune defenses. Biofilm-associated cells actively release extracellular vesicles that contribute to extracellular matrix assembly, enhance biofilm maturation, and promote resistance to antifungal drugs [131]. Some recent studies found that Coronaviruses such as, Murine Hepatitis Virus (MHV) and SARS-CoV-2 are able to persist within the biofilms creates favorable microenvironments that enhance viral stability, maturation, and infectivity [16,132]. According to another study, SARS-CoV-2 may similarly be sequestered within biofilms at multiple anatomical sites, including the oral cavity, bronchoalveolar necrotic tissue, and fibrotic lung tissue. Prolonged viral persistence within these biofilm niches has the potential to contribute to sustained tissue injury, progressive necrosis, and the formation of cavitary lung lesions [133].

## 10. Diagnosis of Fungal Co-Infections in COVID-19 Patients

The early identification of *Aspergillus* and *Candida* infections in COVID-19 patients can be facilitated using various diagnostic methods, such as histopathology, direct microscopic examination, specimen culture, 1,3-β-D-glucan test, galactomannan (cell wall constituent of fungi) test, and polymerase chain reaction (PCR)-based assays, enabling the implementation of effective therapeutic strategies [95]. The diagnostic methods of fungal co-infection during viral outbreaks are described below:

### 10.1. Direct Microscopy and Histopathology

The diagnosis of fungal co-infection is crucial before initiating treatment. Detecting these co-infections has become a significant challenge, particularly in the context of COVID-19 pandemic [134]. Despite the availability of a wide range of techniques, the identification of fungal co-infection requires painstaking effort and challenging examination due to the frequent occurrence of false-positive or false-negative results [117]. Microscopic inspection, histological examination, sample culture, and serological and molecular assays are commonly used to diagnose fungal infections. Direct microscopic examination enables quick and superficial detection of fungi [22,95]. It is commonly performed on specimens like sputum, bronchoalveolar lavage fluid, tracheal aspirates, tissue biopsies, and other clinical samples. Its major advantage is the rapid visualization of fungal elements from all types of fungal pathogens; however, its sensitivity depends on fungal burden, specimen quality, and examiner expertise and species-level identification is generally not possible [135]. A microscopic detection system would be accurate if followed by serological tests or molecular assays, such as PCR [136]. Although microscopic images offer rapid detection and moderate specificity, they often provide ambiguous clues for hyphal detection and demonstrate inconsistent reliability due to variations in the skills of the individuals performing the examination [15].

Histopathological examination stands as a primary choice for the efficient detection of invasive *Aspergillus* in virus-infected patients such as those with COVID-19. Histopathological examination is typically performed on tissue biopsy specimens. Its major advantage lies in confirming invasive disease and differentiating colonization from infection; however, obtaining tissue samples can be invasive and may not always be feasible in critically ill patients. This approach is particularly valuable for invasive aspergillosis and mucormycosis [137]. This method employs different fungal strains, namely, Grocott–Gomori’s methenamine-silver stain and periodic acid–Schiff stains, which play a crucial role in staining the branch hyphae of *Aspergillus* species in suspected patients [138]. Unfortunately, *Aspergillus* cannot be discerned in the presence of other filamentous fungi, such as *Fusarium* spp. and *Scedosporium* spp. However, microscopic examination before detection may bolster the efficacy of this detection system [95]. By contrast, CT scans have been used for fungal infection detections in COVID-19 patients, primarily to visualize destructive physiological changes [139].

### 10.2. Biomarker Based Detection

The immunoassay method employing a monoclonal antibody 2DA6 can efficiently detect only the presence of α-1,6-linked mannose of *Zygomycota* and *Ascomycota* [140]. The lateral flow immunoassay is more practical than the enzyme-linked immunosorbent assay (ELISA); however, this method may not effectively detect a variety of fungal infections because of the specificity of the employed monoclonal antibody for the conserved part of certain fungal species [141]. ELISA has demonstrated exceptional sensitivity and specificity for identifying fungal infections caused by *Scedosporium* and *Lomentospora* spp. in patients with cystic fibrosis. This ELISA method was developed to detect immunoglobulin G in the serum of cystic fibrosis patients using whole-cell protein preparations from *Scedosporium boydii* [142]. This enzyme immunoassay is highly effective in detecting blastomycosis antigens in urine, with sensitivity and specificity of >90%. Blastomycosis is a fungal infection of the lungs caused by *Blastomyces dermatitidis* [143]. Serum or plasma samples are commonly used for 1,3-β-D-glucan testing. 1,3-β-D-glucan is a common component of invasive fungal cell wall, especially *Aspergillus* that can be detected in infected individual [144]. Therefore, assessing blood 1,3-β-D-glucan (BG) antigens might be one of the optimal diagnostic techniques for detecting COVID-19-associated invasive aspergillosis since this biomarker was positive in 13 out of 15 patients with confirmed CAPA [136]. Galactomannan, another primary component of fungal cell walls, is frequently released into the bronchoalveolar lavage fluid by the growing hyphae of *Aspergillus* species. Bronchoalveolar lavage fluid and serum are preferred for galactomannan detection. The detection of galactomannan in bronchoalveolar lavage fluid yielded 100% sensitivity and 76.2% specificity, with subtle or no differences compared with those of radiological assays [106]. Therefore, the abovementioned serological assay can be suggested for COVID-19 patients for the early identification of fungal co-infections.

### 10.3. Molecular Diagnostic Methods

Molecular methods, such as PCR and real-time PCR, can detect 18s rRNA genes or internal transcribed spacers (ITS) in the order of fungi, such as *Mucorales* [140]. Unfortunately, they are not as effective in recognizing specific fungi, such as *Mucormycetes*, and their drawbacks are linked to limited clinical evaluation. *Cryptococcus* in fungal co-infections can be detected using molecular diagnostic tools, such as pan-fungal PCR, multiplex PCR, isothermal amplification, and probe-based microarrays [95]. PCR-based diagnosis provides superior results compared with galactomannan immunoassays for the early diagnosis of COVID-19-associated pulmonary aspergillosis [145]. PCR-based methods are particularly useful for detecting *Aspergillus*, *Candida*, *Cryptococcus*, *Mucorales*, and *P. jirovecii* infections. Multiplex PCR-based detection is one of the most effective diagnostic methods for fungal species such as *Candida* spp. [146]. The specificity of the method was 97.81%, which was consistent with the results obtained from direct examination and Giemsa staining. The *Aspergillus*-specific PCR and the *Aspergillus*-specific lateral flow devices test showed high efficacy in detecting CAPA, accentuating the importance of exploiting molecular approaches for fungal co-infection detection [22]. Molecular assays are generally performed using blood, serum, bronchoalveolar lavage fluid, respiratory secretions, or tissue specimens depending on the suspected fungal pathogen. Their major advantages include high sensitivity, rapid turnaround time, and species-level identification. However, limitation include limited availability in resource-constrained settings [147].

The BG assay coupled with PCR offers high specificity for the early detection of *P. jirovecii*, which can lead to high morbidity in patients with COVID-19 [148]. The detection of *P. jirovecii* is highly challenging. This predominant fungus is found in many co-infectious agents predominantly associated with respiratory diseases. However, *P. jirovecii* can be efficiently detected by next-generation sequencing, which eliminates all the current shortcomings in diagnosis. This robust diagnostic system may be a promising technology for the detection of fungal co-infections [149]. Next-generation sequencing has also been used for *Mucorales* diagnosis by sequencing the ITS region [16]. Matrix-assisted laser desorption ionization time-of-flight mass spectrometry (MALDI-TOF) has earned plaudit for its robust detection and well-established protocol. MALDI-TOF MS is primarily applied to positive culture isolates and blood culture specimens. Its major advantage is the rapid and accurate identification of fungal species. It takes only 30 min to identify *C. albicans*, with a sensitivity of 95.9%, and NAC, with a sensitivity of 86.5%, from positive blood culture bottles, eliminating the necessity for subculturing [150]. Despite the limitations of the MALDI-TOF MS technique related to in-house database problems, it appears to be a promising diagnostic tool for detecting fungal co-infections [151].

For the detection of fungal co-infections, large-scale studies are imperative for the identification of early biomarkers. Furthermore, the optimization of diagnostic methods must be established based on population and geographical variations in disease prevalence and disposition. Ultimately, the medical sector must be equipped to detect co-infections promptly during future viral outbreaks to ensure effective treatment. Delays in the diagnosis of co-infections, such as severe infections caused by *Aspergillus*, *Candida*, and *Rhizopus* species, could lead to delayed administration of effective antifungal therapy, resulting in high morbidity and mortality.

## 11. Treatment for Fungal Co-Infections

### 11.1. Standard Antifungal Therapies

Standard antifungal therapies remain the cornerstone of treatment for fungal co-infections, particularly in patients experiencing viral infections that compromise immune defenses or disrupt epithelial barriers. These medications target key components of the fungal cell membrane or cell wall, thereby inhibiting fungal growth or inducing cell death. The five main classes of antifungal drugs approved for clinical use in humans are polyenes, azoles, echinocandins, allylamines, and antimetabolites [152].

**Polyenes:** Polyenes produced by Gram-positive bacteria are macrolides that bind to ergosterol, form pores in the fungal cell membrane, and cause disruption of chemiosmotic gradients and intracellular constituent leakage [153]. Currently, amphotericin B is certified for the treatment in solo or in combination with other agents against certain invasive fungal diseases including aspergillosis, candidiasis, cryptococcosis, and mucormycosis [154]. Amphotericin B treatment is therefore recommended for COVID-19 patients who have severe fungal infections [16]. Amphotericin B, available in two formulations (amphotericin deoxycholate and lipid-based amphotericin B), is a broad-spectrum polyene macrolide widely used in the treat fungal co-infections [155,156]. Aerosolized amphotericin B is effective as a prophylactic measure for lowering the IPA in patients with neutropenia [156]. Invasive aspergillosis observed in COVID-19 was also treated with amphotericin B formulations [157]. The CDC recommends amphotericin B in combination with flucytosine as an initial treatment for CM. For COVID-19-associated mucormycosis, liposomal amphotericin B is recommended as the first-line antifungal therapy, while isavuconazole or posaconazole may be used as an alternative or salvage therapies depending on the clinical circumstances [138].

**Figure 3:**
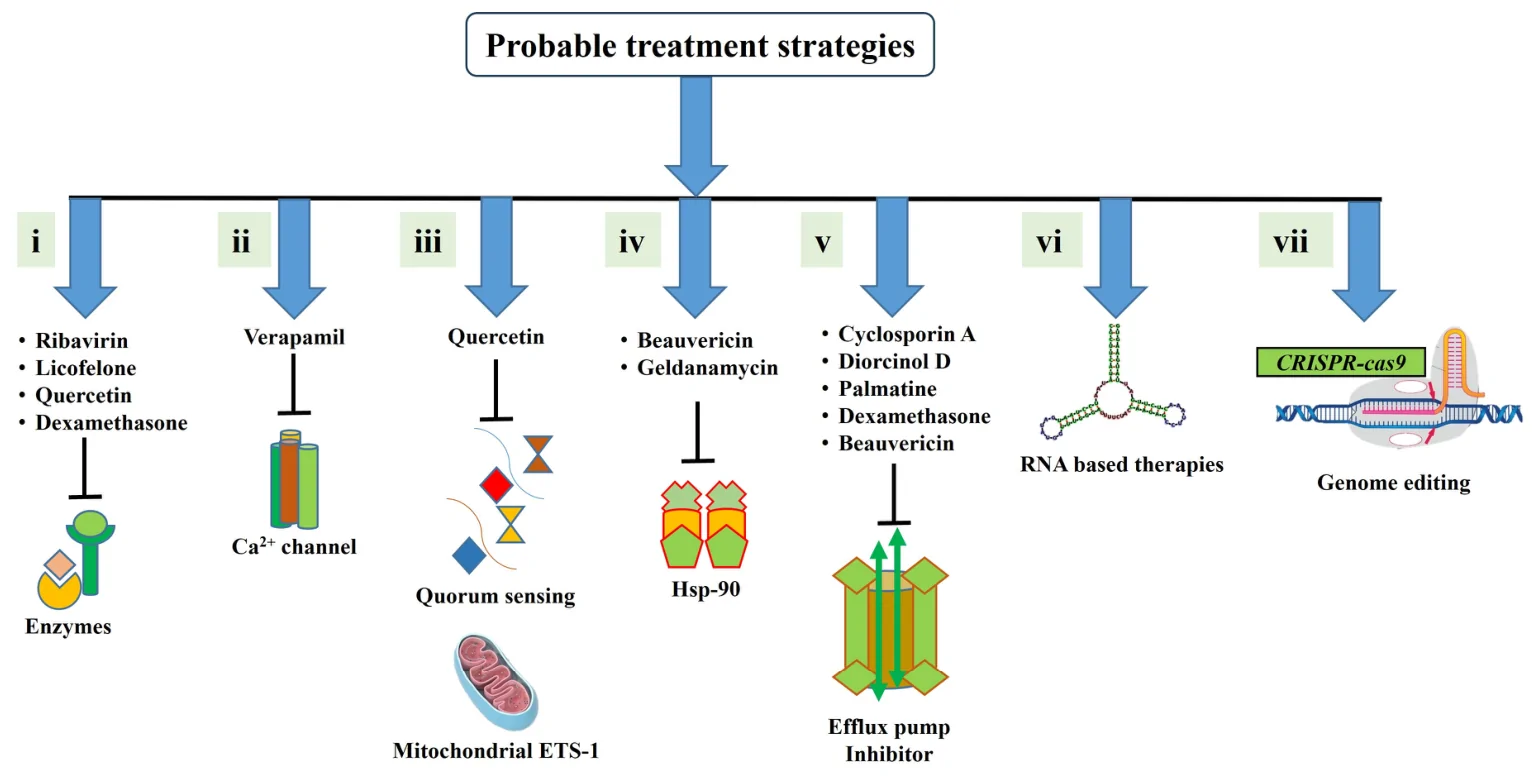
Probable treatment strategies targeting fungal pathogenic mechanisms. A range of therapeutic approaches are illustrated, including (i) enzyme inhibitors, such as ribavirin, licofelone, quercetin, and dexamethasone; (ii) calcium-channel inhibition by verapamil; (iii) disruption of quorum sensing and mitochondrial ETS-1 pathways by quercetin; (iv) inhibition of Hsp90 using beauvericin and geldanamycin; (v) efflux-pump inhibition via cyclosporin A, diorcinol D, palmatine, dexamethasone, and beauvericin; (vi) RNA-based therapeutic strategies; and (vii) genome editing tools, such as CRISPR-Cas9. These approaches collectively represent potential therapeutic avenues for controlling fungal infections.

**Azoles:** Azoles are synthetic antifungals that prevent the synthesis of ergosterol, a major component of fungal plasma membranes, by targeting the enzyme 14α-sterol demethylase [158]. Fluconazole, the first approved antifungal triazole, was effective against *Candida* spp.; however, it was not effective against other fungal copathogenesis [156]. The CDC has also recommended this drug for the treatment of cryptococcal infections. Also, combination therapy that includes administration of more than one drug can be considered in severe cases [49]. Voriconazole and posaconazole are second-generation triazole drugs that are used against infections caused by invasive fungi, for instance, *Aspergillus* and Mucormycetes. While both of the drugs have significant antifungal activities against *Aspergillus* [159], posaconazole, an extended spectrum triazole antifungal, was found to be specifically active against multiple species in the order Mucorales [160]. Other triazoles, such as isavuconazole and itraconazole are also used for treatment; [72,161]. Although triazoles are generally safe for patients, therapeutic drug monitoring is strongly recommended to monitor for potential side effects [162]. According to the recommendations of CDC, triazoles, such as itraconazole, voriconazole, posaconazole, and esaconazole, should be prescribed in combination with corticosteroids for allergic aspergillosis. In the case of invasive aspergillosis, which is observed in COVID-19 patients, the CDC recommends the use of voriconazole, lipid amphotericin B formulations, isavuconazole, itraconazole, and echinococcus (micafungin or caspofungin) (Figure 3) [124].

**Echinocandins:** Echinocandins interfere with the formation of 1,3-β-D-glucan in the fungal cell wall by inhibiting 1,3-β-glucan synthase in a non-competitive manner [163]. Caspofungin and anidulafungin belong to the echinocandin class of antifungal drugs and exhibit fungicidal and fungistatic activities against *Candida* and *Aspergillus* spp., respectively. As the oldest and most commonly used echinocandin, sufficient data on its safety and efficacy are available for caspofungin. Therefore, this drug is the only echinocandin approved for use in children and neonates [156].

**Allylamines and Antimetabolites:** Allylamines function as ergosterol biosynthesis inhibitors of squalene epoxidase, causing sterol depletion and accumulation of the squalene precursor of the enzyme, which in turn disrupts membrane structures and functions, such as nutrient uptake. Among allylamines, terbinafine is effective against *C. albicans* [164]. Antimetabolites, such as 5-flucytosine, have been used to combat cryptococcal infections caused by *C. neoformans* and *C. gattii* [165]. This compound interferes with fungal DNA and RNA synthesis after intracellular conversion to fluorinated pyrimidines and are commonly used in combination therapy to enhance efficacy and reduce resistance.

### 11.2. Therapies Targeting Biofilms

Biofilm formation represents a major obstacle in the treatment of fungal diseases, as biofilms contribute to nearly 80% of all microbial infections in humans and are a leading cause of therapeutic failure and infection recurrence [16]. There is substantial evidence demonstrating that many diseases caused by *Aspergillus*, including aspergilloma and IPA, as well as infections caused by *C. albicans* are predominantly mediated by biofilm formation [166,167]. Azoles are commonly prescribed for the treatment of cutaneous fungal infections and vulvovaginal candidiasis owing to their low toxicity and relative affordability. Nevertheless, the antibiofilm action of azoles is weak, and resistance to azoles has been noted in numerous studies [168,169]. Among the available antifungal drugs, only echinocandins and liposomal formulations of amphotericin B have a strong ability to treat biofilm-based fungal infections [170,171]. Echinocandins mainly inhibit the formation of cell walls by inhibiting 1,3-β-glucan synthase that synthesize a major constituent of the fungal cell wall named 1,3-β-D-glucan [163]. Amphotericin B attaches to ergosterol, the main sterol in fungal membranes, which disrupts membrane function and allows cellular contents to seep out rather than block an enzyme [172,173].

Antifungal therapies, such as amphotericin B or isavuconazole, alone or in combination with posaconazole, have remained the basis for treating mucormycosis [16,174]. The presence of elevated accessible serum iron content in the host affected by mucormycosis suggests that host iron availability plays a significant role as a major pathogenic factor. Mucorales fungi exploit this increased iron availability, leading to rapid growth and spread of the infection [175]. Lactoferrin, a natural glycoprotein, can act as an iron chelator; thus, it can be applied as a potential fungistatic agent to treat mucormycosis [106]. In addition to lactoferrins, several drugs that are not conventional antifungal agents have been used. For example, the antiviral drug ribavirin functions by inhibiting viral RNA-dependent RNA polymerase to alleviate the symptoms of COVID-9 patients with serious conditions [176]. This antiviral agent also displayed antifungal activity against fluconazole-resistant *C. albicans* [176]. Licofelon, an inhibitor of cyclooxygenase and lipoxygenase, in combination with fluconazole can prevent biofilm formation by *C. albicans* [177]. Naturally occurring flavonoid quercetins exhibit a broad spectrum of antifungal properties, which arise from their ability to downregulate the genes responsible for biofilm formation in *C. albicans* [178]. Licofelon, ribavirin, and quercetins mainly affect fungal enzymes; therefore, they inhibit metabolic processes as well as the formation and function of the cell wall and cell membrane. Verapamil, a calcium channel blocker, is a potent antifungal agent against NAC [179]. Beauvericin, a fungus-derived bioactive compound, acts as an antifungal agent against azole-resistant *Candida* isolates by inhibiting multidrug efflux [180]. Diorcinol D, an endolichenic fungal compound, exhibits fungicidal activity by destroying the cytoplasmic membrane and promoting ROS accumulation [181]. In general, these drugs target various crucial components for fungal survival.

### 11.3. Emerging Therapeutic Approaches

The pandemic has opened a door to evaluate the efficiency of certain emerging therapeutics as an alternative approach to the conventional one. Among the various approaches, the newly deduced mRNA vaccine has already been commercialized and some of the antibody based therapeutic strategies have got the FDA approval. However, CRISPR/Cas based therapeutics are still under experiment with some evidence to have potential to be used in future pandemic.

#### 11.3.1. Vaccine and Antibody-Based Therapeutic Options

Vaccination against SARS-CoV-2 has played a pivotal role in limiting viral transmission and preventing severe and fatal disease outcomes. However, despite the growing body of evidence supporting vaccine effectiveness at the population level, data specifically addressing its impact among hospitalized COVID-19 patients remain comparatively limited. The risk of mortality, ICU admission, and duration of hospitalization in hospitalized COVID-19 patients stratified by vaccination status. Full vaccination against SARS-CoV-2 is associated with significantly improved clinical outcomes, including reduced mortality, lower rates of ICU admission, and shorter hospital stays, underscoring the continued importance of vaccination even among patients who require hospital-based care [182,183]. Consequently, SARS-CoV-2 vaccination may indirectly reduce the risk of COVID-19-associated fungal infections such as CAPA and CAM [184].

Antibody-based therapies represent an important therapeutic approach for the prevention and management of SARS-CoV-2 infection, particularly among individuals at high risk of progression to severe disease. Neutralizing antibodies (nAbs) are designed to recognize specific epitopes on the SARS-CoV-2 spike protein, membrane protein and nucleocapsid protein particularly the receptor-binding domain (RBD), thereby blocking viral attachment and entry into host cells [185]. Several monoclonal antibodies have demonstrated clinical efficacy in treating SARS-CoV-2 and some of them has obtained FDA approval for clinical application. The U.S. FDA granted emergency authorization for the monoclonal antibody combinations—bamlanivimab with etesevimab and casirivimab with imdevimab for the treatment of COVID-19 patients [186]. However, the emergence of antigenically distinct SARS-CoV-2 variants has substantially reduced or eliminated the activity of many previously authorized antibodies [187]. Consequently, the clinical utility of anti-SARS-CoV-2 mAbs is highly dependent on the susceptibility of circulating variants and the timing of administration. Success has also been obtained in treating COVID-19 by introducing a novel treatment method combining antiviral remdesivir and neutralizing antibody sotrovimab [188].

Convalescent plasma therapy involves the transfusion of plasma collected from individuals who have recovered from COVID-19 and contains antibodies directed against SARS-CoV-2. The therapeutic rationale is based on the passive transfer of neutralizing antibodies, which may facilitate viral clearance and limit disease progression, particularly in patients who are unable to produce an effective endogenous humoral immune response. A meta-analysis found that patients treated with convalescent plasma had a 26% lower risk of requiring hospitalization. Subgroup analysis further indicated an 8% reduction in the risk of progression to ICU-level disease among patients with COVID-19 who received convalescent plasma [189]. In the context of fungal co-infections, convalescent plasma may have an indirect therapeutic benefit by promoting viral clearance and potentially reducing prolonged viral replication and severe pulmonary disease [190].

#### 11.3.2. RNA-Based Therapeutics

RNA-based therapeutics, such as CRISPR/Cas 13 systems have opened new possibilities for therapeutic interventions by enabling precise editing or regulation of genetic targets [191,192]. Studies on model organisms have yielded abundant information regarding RNA regulation in fungi, and this type of therapy may represent a novel strategy for treating human fungal infections. One potential target for RNAi technology is RNA regulatory systems that discriminate between pathogens and non-pathogens [191]. However, a comprehensive framework for RNA-based therapy for human fungal diseases has not yet been fully developed due to the occurrence of several complications. First, the determination of suitable targets for RNA-based therapeutics to specifically suppress the fungal pathogen without negatively affecting the host or commensal species is needed. Second, the delivery of antifungal RNAs to specific infected locations is another challenge because of the diverse cell walls and capsule structures of fungal pathogens. Progress in the development of RNA therapeutics may soon lead to their use in human fungal pathogen therapy. Although a comprehensive strategy for CRISPR/Cas-mediated antifungal therapy has not yet been defined, the available results support the possibility of developing a successful method for treating fungal diseases [193]. Further research is required to identify new and effective CRISPR/Cas-mediated antifungal therapies to combat these pathogenic fungi. The presence and persistence of drug resistance in fungal species implies that novel therapeutic drugs will be required in the near future to effectively treat invasive fungal infections [68].

### 11.4. Antifungal Stewardship

Antifungal stewardship is an important component of the management of COVID-19-associated fungal co-infections. Although empirical antifungal therapy may be warranted in critically ill patients with a high clinical suspicion of invasive fungal infection, unnecessary or prolonged antifungal exposure can increase drug-related toxicity, healthcare costs, drug–drug interactions, and the evolution of antifungal-resistant organisms [194]. Therefore, rapid and accurate diagnosis is essential for distinguishing fungal colonization from invasive disease and for enabling pathogen-directed treatment.

## 12. Conclusions and Future Perspective

COVID-19-associated fungal co-infections remain a major clinical challenge because of their difficult diagnosis, rapid progression, complex host–pathogen interactions, and substantial morbidity and mortality. Although advances in diagnostic approaches have improved fungal detection, important gaps remain in identifying reliable biomarkers that can distinguish colonization from invasive disease and enable diagnosis at an early stage. The establishment of standardized and clinically validated diagnostic criteria in healthcare settings is also urgently needed. Furthermore, the optimal timing for initiating antifungal therapy, the selection and duration of antifungal regimens, and the role of combination therapies require further investigation. At the mechanistic level, greater understanding of how SARS-CoV-2-induced immune dysregulation, epithelial and endothelial barrier damage, outcome of corticosteroid and immunomodulatory therapies, and the role of other host factors in impairment of antifungal immunity may provide new opportunities for targeted interventions. The emergence of antifungal resistance, particularly among Aspergillus, Candida, and Mucorales, further emphasizes the need for systematic surveillance and improved antifungal stewardship. Future research should therefore prioritize the development and validation of early diagnostic biomarkers, prospective studies to define optimal antifungal treatment strategies, and investigation of novel antifungal therapies. Addressing these specific knowledge gaps will be essential for enabling earlier diagnosis and individualized treatment for patients with COVID-19-associated fungal co-infections.

## Figures and Tables

**Table 1:** Spectrum of fungal co-pathogens and antifungal treatments in COVID-19 and other viral diseases.

Primary Viral Disease	Invasive Fungal Infection	Fungal Co-Pathogen	Drug Used	References
SARS-CoV-2	Candidemia, Systemic candidiasis	*C. auris*, *C. tropicalis*, *C. albicans*, *C. parapsilosis*, *C. orthopsilosis*, *C. glabrata*	Fluconazole, caspofungin, voriconazole	[70]
SARS-CoV-2	Aspergillosis	*A. fumigatus*	Voriconazole, ISA, caspofungin 5	[71]
SARS-CoV-2	Aspergillosis	*A. fumigatus*	Voriconazole, voriconazole + ISA	[72]
SARS-CoV-2	Candidiasis	*C. glabrata*, *C. albicans*	Not reported	[73]
SARS-CoV-2	Aspergillosis	*A. fumigatus*, *Aspergillus* spp.	Voriconazole + anidulafungin, amphotericin (liposomal)	[74]
SARS-CoV-2	Candidiasis	*C. auris*, *C. albicans*, *C. krusei*, *C. tropicalis*	Fluconazole, voriconazole, posaconazole, ISA, 5-flucytosine, caspofungin 5, MFG, anidulafungin, and amphotericin.	[75]
SARS-CoV-2	Candidiasis/Aspergillosis	*Candida* spp., *Aspergillus* spp.	Not reported	[76]
SARS-CoV-2	Invasive pulmonary mucormycosis	*Rhizopus microsporus*	Voriconazole	[77]
SARS-CoV-2	Invasive aspergillosis	*A. flavus*, *A. fumigatus*, *C. albicans*	Remdesivir and voriconazole	[78]
SARS-CoV-2	Mucormycosis	*R. microsporus*; *R. arrhizus*; *Mucor* spp.	Amphotericin, ISA, voriconazole and posaconazole	[79]
SARS-CoV-2	Mucormycosis	*Rhizopus* spp.	Amphotericin	[80]
SARS-CoV-2	Mucormycosis	-	Amphotericin	[81]
SARS-CoV-2	Mucormycosis	*Rhizopus* spp.	Amphotericin, posaconazole	[82]
SARS-CoV-2	Mucormycosis	*Mucor* spp.	Amphotericin, amphotericin + oral posaconazole	[83]
SARS-CoV-2	Mucormycosis	*R. azygosporus*	Posaconazole + isavuconazole	[84]
SARS-CoV-2	Aspergillosis	*A. flavus*, *A. niger*, *A. fumigatus*	Amphotericin	[85]
SARS-CoV-2	Mucormycosis, Aspergillosis	-	Lip amphotericin	[86]
Influenza A	Aspergillosis	*A. fumigatus*, *Aspergillus* spp.	Voriconazole, voriconazole + echinocandin	[87]
	Aspergillosis	*A. fumigatus*, *Aspergillus* spp.	Voriconazole	[62]
H1N1	Disseminated mucormycosis	-	Amphotericin	[88]
H1N1	Mucormycosis	*Lichtheimia* spp.	Lip amphotericin + posaconazole	[89]
H1N1	Tracheal mucormycosis	-	Amphotericin	[90]
Influenza A and Influenza B	Invasive aspergillosis	*Aspergillus* spp.	Not reported	[91]
Influenza A	Invasive aspergillosis	*A. fumigatus*	Voriconazole	[61]
Influenza A	Invasive pulmonary aspergillosis	*A. fumigatus*	Amphotericin + anidulafungin	[92]
			Posaconazole, amphotericin + voriconazole + caspofungin 5	
			Voriconazole	
			Voriconazole	
Influenza A	Invasive pulmonary aspergillosis	*A. fumigatus*, *Aspergillus* spp.	Voriconazole, voriconazole + anidulafungin, amphotericin	[58]
Influenza A			Voriconazole, voriconazole + amphotericin, voriconazole + micafungin, voriconazole+ caspofungin 5, amphotericin	
Influenza B			Voriconazole	
Influenza A	Invasive aspergillosis	*Aspergillus* spp.	Amphotericin, fluconazole + amphotericin, fluconazole + voriconazole, amphotericin + voriconazole + micafungin, voriconazole + caspofungin 5 + micafungin + amphotericin	[64]
Influenza A			Fluconazole, voriconazole	
Influenza B			Voriconazole, itraconazole + amphotericin, voriconazole + micafungin	
Influenza A	Aspergillosis	*Aspergillus* spp.	Voriconazole	[93]
Influenza A	Invasive aspergillosis	*A. fumigatus*	Voriconazole	[94]

## Data Availability

No new data were created or analyzed in this study. Data sharing is not applicable to this article.

## References

[1] Piret J., Boivin G. (2021). Pandemics Throughout History. Front. Microbiol..

[2] Baker R.E., Mahmud A.S., Miller I.F., Rajeev M., Rasambainarivo F., Rice B.L., Takahashi S., Tatem A.J., Wagner C.E., Wang L.F. (2022). Infectious Disease in an Era of Global Change. Nat. Rev. Microbiol..

[3] Chippaux J.P., Chippaux A. (2018). Yellow Fever in Africa and the Americas: A Historical and Epidemiological Perspective. J. Venom. Anim. Toxins Incl. Trop. Dis..

[4] Trovato M., Sartorius R., D’Apice L., Manco R., De Berardinis P. (2020). Viral Emerging Diseases: Challenges in Developing Vaccination Strategies. Front. Immunol..

[5] Raihan T., Rabbee M.F., Roy P., Choudhury S., Baek K.H., Azad A.K. (2021). Microbial Metabolites: The Emerging Hotspot of Antiviral Compounds as Potential Candidates to Avert Viral Pandemic Alike COVID-19. Front. Mol. Biosci..

[6] WHO (2026). Overall Global Risk and Confidence Overall Risk Statement.

[7] Calderón-Parra J., Mills-Sanchez P., Moreno-Torres V., Tejado-Bravo S., Romero-Sánchez I., Balandin-Moreno B., Calvo-Salvador M., Portero-Azorín F., García-Masedo S., Muñez-Rubio E. (2022). COVID-19-Associated Pulmonary Aspergillosis (CAPA): Risk Factors and Development of a Predictive Score for Critically Ill COVID-19 Patients. Mycoses.

[8] Barman T.K., Metzger D.W. (2021). Disease Tolerance during Viral-Bacterial Co-Infections. Viruses.

[9] Shah T., Shah Z., Yasmeen N., Baloch Z., Xia X. (2022). Pathogenesis of SARS-CoV-2 and *Mycobacterium tuberculosis* Coinfection. Front. Immunol..

[10] Aunkor M.T.H., Raihan T., Prodhan S.H., Metselaar H.S.C., Malik S.U.F., Azad A.K. (2020). Antibacterial Activity of Graphene Oxide Nanosheet against Multidrug Resistant Superbugs Isolated from Infected Patients. R. Soc. Open Sci..

[11] Salazar F., Bignell E., Brown G.D., Cook P.C., Warris A. (2022). Pathogenesis of Respiratory Viral and Fungal Coinfections. Clin. Microbiol. Rev..

[12] Rawson T.M., Moore L.S.P., Zhu N., Ranganathan N., Skolimowska K., Gilchrist M., Satta G., Cooke G., Holmes A. (2020). Bacterial and Fungal Coinfection in Individuals with Coronavirus: A Rapid Review to Support COVID-19 Antimicrobial Prescribing. Clin. Infect. Dis..

[13] Hakim A., Hasan M.M., Hasan M., Lokman S.M., Azim K.F., Raihan T., Chowdhury P.A., Azad A.K. (2021). Major Insights in Dynamics of Host Response to SARS-CoV-2: Impacts and Challenges. Front. Microbiol..

[14] Khodavaisy S., Sarrafnia H., Abdollahi A. (2024). Outcomes of Patients with COVID-19 and Fungal Coinfections: A Systematic Review and Meta-Analysis Study. Iran. J. Pathol..

[15] Xiang Z., Koo H., Chen Q., Zhou X., Liu Y., Simon-Soro A. (2021). Potential Implications of SARS-CoV-2 Oral Infection in the Host Microbiota. J. Oral Microbiol..

[16] Naveen K.V., Saravanakumar K., Sathiyaseelan A., MubarakAli D., Wang M.-H.H. (2022). Human Fungal Infection, Immune Response, and Clinical Challenge—A Perspective During COVID-19 Pandemic. Appl. Biochem. Biotechnol..

[17] Martins-Filho P.R., Tavares C.S.S., Santos V.S. (2020). Factors Associated with Mortality in Patients with COVID-19. A Quantitative Evidence Synthesis of Clinical and Laboratory Data. Eur. J. Intern. Med..

[18] Hoenigl M., Seidel D., Sprute R., Cunha C., Oliverio M., Goldman G.H., Ibrahim A.S., Carvalho A. (2022). COVID-19-Associated Fungal Infections. Nat. Microbiol..

[19] Mayer F.L., Wilson D., Hube B. (2013). *Candida albicans* Pathogenicity Mechanisms. Virulence.

[20] Valencia-Ledezma O.E., Reyes-Montes M.d.R., Acosta-Altamirano G., Frías-De-León M.G., García-Salazar E., Duarte-Escalante E., Santiago-Abundio J., González-Miguel Z., García-Hernández M.d.L., Martínez-Quezada R. (2025). Invasive Candidiasis Coinfection in Patients with Severe COVID-19 Disease: Scoping Review. Pathogens.

[21] Segrelles-Calvo G., de S Araújo G.R., Llopis-Pastor E., Carrillo J., Hernández-Hernández M., Rey L., Melean N.R., Escribano I., Antón E., Zamarro C. (2021). *Candida* Spp. Co-Infection in COVID-19 Patients with Severe Pneumonia: Prevalence Study and Associated Risk Factors. Respir. Med..

[22] Borman A.M., Palmer M.D., Fraser M., Patterson Z., Mann C., Oliver D., Linton C.J., Gough M., Brown P., Dzietczyk A. (2021). COVID-19-Associated Invasive Aspergillosis: Data from the UK National Mycology Reference Laboratory. J. Clin. Microbiol..

[23] Singh A., Singh J., Kumar S. (2025). Aspergillosis: A Comprehensive Review of Pathogenesis, Drug Resistance, and Emerging Therapeutics. J. Food Drug Anal..

[24] Xiang H., Zhang L., Cai M., Zhang Y. (2025). Insights into Dysregulated Innate Immunity in the Pathogenesis of COVID-19-Associated Pulmonary Aspergillosis. Infection.

[25] Prakash H., Chakrabarti A. (2021). Epidemiology of Mucormycosis in India. Microorganisms.

[26] Silva D.L., Peres N.T.A., Santos D.A. (2025). Key Fungal Coinfections: Epidemiology, Mechanisms of Pathogenesis, and Beyond. mBio.

[27] Rammaert B., Lanternier F., Zahar J.R., Dannaoui E., Bougnoux M.E., Lecuit M., Lortholary O. (2012). Healthcare-Associated Mucormycosis. Clin. Infect. Dis..

[28] Foster C.E., Revell P.A., Campbell J.R., Marquez L. (2021). Healthcare-Associated Pediatric Cutaneous Mucormycosis at Texas Children’s Hospital, 2012–2019. Pediatr. Infect. Dis. J..

[29] García-Carnero L.C., Mora-Montes H.M. (2022). Mucormycosis and COVID-19-Associated Mucormycosis: Insights of a Deadly but Neglected Mycosis. J. Fungi.

[30] Mahalaxmi I., Jayaramayya K., Venkatesan D., Subramaniam M.D., Renu K., Vijayakumar P., Narayanasamy A., Gopalakrishnan A.V., Kumar N.S., Sivaprakash P. (2021). Mucormycosis: An Opportunistic Pathogen during COVID-19. Environ. Res..

[31] Esher S.K., Zaragoza O., Alspaugh J.A. (2018). Cryptococcal Pathogenic Mechanisms: A Dangerous Trip from the Environment to the Brain. Mem. Inst. Oswaldo Cruz.

[32] Del Prete V., Paterno G., Cennamo O., Berrilli F., Di Cave D. (2023). The Effect of COVID-19 on the Frequency of *Pneumocystis jirovecii* Pneumonia: A Monocentric, Retrospective, and Observational Study. BMC Infect. Dis..

[33] Apostolopoulou A., Fishman J.A. (2022). The Pathogenesis and Diagnosis of *Pneumocystis jiroveci* Pneumonia. J. Fungi.

[34] Xu J.-B., Guan W.-J., Zhang Y.-L., Qiu Z.-E., Chen L., Hou X.-C., Yue J., Zhou Y.-Y., Sheng J., Zhao L. (2024). SARS-CoV-2 Envelope Protein Impairs Airway Epithelial Barrier Function and Exacerbates Airway Inflammation via Increased Intracellular Cl− Concentration. Signal Transduct. Target. Ther..

[35] Lionakis M.S., Drummond R.A., Hohl T.M. (2023). Immune Responses to Human Fungal Pathogens and Therapeutic Prospects. Nat. Rev. Immunol..

[36] Croft C.A., Culibrk L., Moore M.M., Tebbutt S.J. (2016). Interactions of *Aspergillus fumigatus* Conidia with Airway Epithelial Cells: A Critical Review. Front. Microbiol..

[37] Dimitrov D.S. (2004). Virus Entry: Molecular Mechanisms and Biomedical Applications. Nat. Rev. Microbiol..

[38] Kanta S., Siddhartha M. (2020). Respiratory Virus Infections: Understanding COVID-19. Immunity.

[39] Ziegler C.G.K., Allon S.J., Nyquist S.K., Mbano I.M., Miao V.N., Tzouanas C.N., Cao Y., Yousif A.S., Bals J., Hauser B.M. (2020). SARS-CoV-2 Receptor ACE2 Is an Interferon-Stimulated Gene in Human Airway Epithelial Cells and Is Detected in Specific Cell Subsets across Tissues. Cell.

[40] Pittet L.A., Hall-Stoodley L., Rutkowski M.R., Harmsen A.G. (2010). Influenza Virus Infection Decreases Tracheal Mucociliary Velocity and Clearance of Streptococcus Pneumoniae. Am. J. Respir. Cell Mol. Biol..

[41] Chua R.L., Lukassen S., Trump S., Hennig B.P., Wendisch D., Pott F., Debnath O., Thürmann L., Kurth F., Völker M.T. (2020). COVID-19 Severity Correlates with Airway Epithelium–Immune Cell Interactions Identified by Single-Cell Analysis. Nat. Biotechnol..

[42] Sheppard D.C. (2011). Molecular Mechanism of *Aspergillus fumigatus* Adherence to Host Constituents. Curr. Opin. Microbiol..

[43] Sprague J.L., Schille T.B., Allert S., Trümper V., Lier A., Großmann P., Priest E.L., Tsavou A., Panagiotou G., Naglik J.R. (2024). *Candida albicans* Translocation through the Intestinal Epithelial Barrier Is Promoted by Fungal Zinc Acquisition and Limited by NFκB-Mediated Barrier Protection. PLoS Pathog..

[44] Netea M.G., Joosten L.A.B., Van Der Meer J.W.M., Kullberg B.J., Van De Veerdonk F.L. (2015). Immune Defence against *Candida* Fungal Infections. Nat. Rev. Immunol..

[45] Morton C.O., Griffiths J.S., Loeffler J., Orr S., White P.L. (2022). Defective Antifungal Immunity in Patients with COVID-19. Front. Immunol..

[46] Liu J., Li S., Liu J., Liang B., Wang X., Wang H., Li W., Tong Q., Yi J., Zhao L. (2020). Longitudinal Characteristics of Lymphocyte Responses and Cytokine Profiles in the Peripheral Blood of SARS-CoV-2 Infected Patients. eBioMedicine.

[47] Mina S., Yaakoub H., Annweiler C., Dubée V., Papon N. (2022). COVID-19 and Fungal Infections: A Double Debacle. Microbes Infect..

[48] Manfiolli A.O., dos Reis T.F., de Assis L.J., de Castro P.A., Silva L.P., Hori J.I., Walker L.A., Munro C.A., Rajendran R., Ramage G. (2018). Mitogen Activated Protein Kinases (MAPK) and Protein Phosphatases Are Involved in *Aspergillus fumigatus* Adhesion and Biofilm Formation. Cell Surf..

[49] Firacative C. (2020). Invasive Fungal Disease in Humans: Are We Aware of the Real Impact?. Mem. Inst. Oswaldo Cruz.

[50] Arastehfar A., Carvalho A., van de Veerdonk F.L., Jenks J.D., Koehler P., Krause R., Cornely O.A., Perlin D.S., Lass-Flörl C., Hoenigl M. (2020). COVID-19 Associated Pulmonary Aspergillosis (CAPA)—From Immunology to Treatment. J. Fungi.

[51] Peng L., Xu Z., Huo Z., Long R., Ma L. (2018). New Insights into the Clinical Characteristics and Prognostic Factors of Pulmonary Fungal Infections from a Retrospective Study in Southwestern China. Infect. Drug Resist..

[52] Tolle L.B., Standiford T.J. (2013). Danger-Associated Molecular Patterns (DAMPs) in Acute Lung Injury. J. Pathol..

[53] Prattes J., Wauters J., Giacobbe D.R., Salmanton-García J., Maertens J., Bourgeois M., Reynders M., Rutsaert L., Van Regenmortel N., Lormans P. (2022). Risk Factors and Outcome of Pulmonary Aspergillosis in Critically Ill Coronavirus Disease 2019 Patientsda Multinational Observational Study by the European Confederation of Medical Mycology. Clin. Microbiol. Infect..

[54] Badiee P., Kordbacheh P., Alborzi A., Malekhoseini S.A., Ramzi M., Mirhendi H., Mahmoodi M., Shakiba E. (2009). Study on Invasive Fungal Infections in Immunocompromised Patients to Present a Suitable Early Diagnostic Procedure. Int. J. Infect. Dis..

[55] Giannella M., Lanternier F., Dellière S., Groll A.H., Mueller N.J., Alastruey-Izquierdo A., Slavin M.A. (2025). Invasive Fungal Disease in the Immunocompromised Host: Changing Epidemiology, New Antifungal Therapies, and Management Challenges. Clin. Microbiol. Infect..

[56] Meawed T.E., Ahmed S.M., Mowafy S.M.S., Samir G.M., Anis R.H. (2021). Bacterial and Fungal Ventilator Associated Pneumonia in Critically Ill COVID-19 Patients during the Second Wave. J. Infect. Public Health.

[57] Bartoletti M., Pascale R., Cricca M., Rinaldi M., Maccaro A., Bussini L., Fornaro G., Tonetti T., Pizzilli G., Francalanci E. (2021). Epidemiology of Invasive Pulmonary Aspergillosis Among Intubated Patients with COVID-19: A Prospective Study. Clin. Infect. Dis..

[58] Waldeck F., Boroli F., Suh N., Wendel Garcia P.D., Flury D., Notter J., Iten A., Kaiser L., Schrenzel J., Boggian K. (2020). Influenza-Associated Aspergillosis in Critically-Ill Patients—A Retrospective Bicentric Cohort Study. Eur. J. Clin. Microbiol. Infect. Dis..

[59] Ezeokoli O.T., Pohl C.H. (2020). Opportunistic Pathogenic Fungal Co-Infections Are Prevalent in Critically Ill COVID-19 Patients: Are They Risk Factors for Disease Severity?. S. Afr. Med. J..

[60] Kubin C.J., McConville T.H., Dietz D., Zucker J., May M., Nelson B., Istorico E., Bartram L., Small-Saunders J., Sobieszczyk M.E. (2021). Characterization of Bacterial and Fungal Infections in Hospitalized Patients with Coronavirus Disease 2019 and Factors Associated with Health Care-Associated Infections. Open Forum Infect. Dis..

[61] Wauters J., Baar I., Meersseman P., Meersseman W., Dams K., De Paep R., Lagrou K., Wilmer A., Jorens P., Hermans G. (2012). Invasive Pulmonary Aspergillosis Is a Frequent Complication of Critically Ill H1N1 Patients: A Retrospective Study. Intensive Care Med..

[62] Van De Veerdonk F.L., Kolwijck E., Lestrade P.P.A., Hodiamont C.J., Rijnders B.J.A., Van Paassen J., Haas P.-J., Dos Santos C.O., Kampinga G.A., Bergmans D.C.J.J. (2017). Influenza-Associated Aspergillosis in Critically Ill Patients. Am. J. Respir. Crit. Care Med..

[63] Perlroth J., Choi B., Spellberg B. (2007). Nosocomial Fungal Infections: Epidemiology, Diagnosis, and Treatment. Med. Mycol..

[64] Shah M.M., Hsiao E.I., Kirsch C.M., Gohil A., Narasimhan S., Stevens D.A. (2018). Invasive Pulmonary Aspergillosis and Influenza Co-Infection in Immunocompetent Hosts: Case Reports and Review of the Literature. Diagn. Microbiol. Infect. Dis..

[65] White P.L., Dhillon R., Cordey A., Hughes H., Faggian F., Soni S., Pandey M., Whitaker H., May A., Morgan M. (2021). A National Strategy to Diagnose Coronavirus Disease 2019-Associated Invasive Fungal Disease in the Intensive Care Unit. Clin. Infect. Dis..

[66] Riche C.V.W., Cassol R., Pasqualotto A.C. (2020). Is the Frequency of Candidemia Increasing in COVID-19 Patients Receiving Corticosteroids?. J. Fungi.

[67] Kimmig L.M., Wu D., Gold M., Pettit N.N., Pitrak D., Mueller J., Husain A.N., Mutlu E.A., Mutlu G.M. (2020). IL-6 Inhibition in Critically Ill COVID-19 Patients Is Associated with Increased Secondary Infections. Front. Med..

[68] Sanguinetti M., Posteraro B., Beigelman-Aubry C., Lamoth F., Dunet V., Slavin M., Richardson M.D. (2019). Diagnosis and Treatment of Invasive Fungal Infections: Looking Ahead. J. Antimicrob. Chemother..

[69] Chen K., Wang Q., Pleasants R.A., Ge L., Liu W., Peng K., Zhai S. (2017). Empiric Treatment against Invasive Fungal Diseases in Febrile Neutropenic Patients: A Systematic Review and Network Meta-Analysis. BMC Infect. Dis..

[70] Duan Y., Ou X., Chen Y., Liang B., Ou X. (2021). Severe Influenza with Invasive Pulmonary Aspergillosis in Immunocompetent Hosts: A Retrospective Cohort Study. Front. Med..

[71] Koehler P., Cornely O.A., Böttiger B.W., Dusse F., Eichenauer D.A., Fuchs F., Hallek M., Jung N., Klein F., Persigehl T. (2020). COVID-19 Associated Pulmonary Aspergillosis. Mycoses.

[72] Rutsaert L., Steinfort N., Van Hunsel T., Bomans P., Naesens R., Mertes H., Dits H., Van Regenmortel N. (2020). COVID-19-Associated Invasive Pulmonary Aspergillosis. Ann. Intensive Care.

[73] Chen N., Zhou M., Dong X., Qu J., Gong F., Han Y., Qiu Y., Wang J., Liu Y., Wei Y. (2020). Epidemiological and Clinical Characteristics of 99 Cases of 2019 Novel Coronavirus Pneumonia in Wuhan, China: A Descriptive Study. Lancet.

[74] van Arkel A.L.E., Rijpstra T.A., Belderbos H.N.A., van Wijngaarden P., Verweij P.E., Bentvelsen R.G. (2020). COVID-19-Associated Pulmonary Aspergillosis. Am. J. Respir. Crit. Care Med..

[75] Chowdhary (2020). Infections in Critically Ill Coronavirus Disease Patients, India, April-July 2020. Emerg. Infect. Dis..

[76] Vijay S., Bansal N., Rao B.K., Veeraraghavan B., Rodrigues C., Wattal C., Goyal J.P., Tadepalli K., Mathur P., Venkateswaran R. (2021). Secondary Infections in Hospitalized COVID-19 Patients: Indian Experience. Infect. Drug Resist..

[77] Zurl C., Hoenigl M., Schulz E., Hatzl S., Gorkiewicz G., Krause R., Eller P., Prattes J. (2021). Autopsy Proven Pulmonary Mucormycosis Due to *Rhizopus microsporus* in a Critically Ill COVID-19 Patient with Underlying Hematological Malignancy. J. Fungi.

[78] Seyedjavadi S.S., Bagheri P., Nasiri M.J., Razzaghi-Abyaneh M., Goudarzi M. (2022). Fungal Infection in Co-Infected Patients with COVID-19: An Overview of Case Reports/Case Series and Systematic Review. Front. Microbiol..

[79] Hoenigl M., Seidel D., Carvalho A., Rudramurthy S.M., Arastehfar A., Gangneux J.P., Nasir N., Bonifaz A., Araiza J., Klimko N. (2022). The Emergence of COVID-19 Associated Mucormycosis: A Review of Cases from 18 Countries. Lancet Microbe.

[80] Revannavar S.M., Supriya P., Samaga L., Vineeth K. (2021). COVID-19 Triggering Mucormycosis in a Susceptible Patient: A New Phenomenon in the Developing World?. BMJ Case Rep..

[81] Mehta S., Pandey A. (2020). Rhino-Orbital Mucormycosis Associated with COVID-19. Cureus.

[82] Mekonnen Z.K., Ashraf D.C., Jankowski T., Grob S.R., Vagefi M.R., Kersten R.C., Simko J.P., Winn B.J. (2021). Acute Invasive Rhino-Orbital Mucormycosis in a Patient with COVID-19-Associated Acute Respiratory Distress Syndrome. Ophthalmic Plast. Reconstr. Surg..

[83] Fumarola B., Signorini L., Lorenzotti S., Lanza P., Saccani B., Van Hauwermeiren E., Mulè A., Piva S., Rota M., Zuccalà F. (2024). Use of nebulized liposomal amphotericin B and posaconazole as antifungal prophylaxis in patients with severe SARS-CoV2 infection in intensive care unit. Infection.

[84] Kanwar A., Jordan A., Olewiler S., Wehberg K., Cortes M., Jackson B.R. (2021). A Fatal Case of Rhizopus Azygosporus Pneumonia Following COVID-19. J. Fungi.

[85] Nasir N., Farooqi J., Mahmood S.F., Jabeen K. (2020). COVID-19-Associated Pulmonary Aspergillosis (CAPA) in Patients Admitted with Severe COVID-19 Pneumonia: An Observational Study from Pakistan. Mycoses.

[86] Moorthy A., Gaikwad R., Krishna S., Hegde R., Tripathi K.K., Kale P.G., Rao P.S., Haldipur D., Bonanthaya K. (2021). SARS-CoV-2, Uncontrolled Diabetes and Corticosteroids—An Unholy Trinity in Invasive Fungal Infections of the Maxillofacial Region? A Retrospective, Multi-Centric Analysis. J. Maxillofac. Oral Surg..

[87] Chen L., Han X., Li Y., Zhang C., Xing X. (2020). Invasive Pulmonary Aspergillosis in Immunocompetent Patients Hospitalised with Influenza A-Related Pneumonia: A Multicenter Retrospective Study. BMC Pulm. Med..

[88] Person B., Bahouth H., Brauner E., Ben-Ishay O., Bickel A., Kluger Y.S. (2010). Surgical Emergencies Confounded by H1N1 Influenza Infection—A Plea for Concern. World J. Emerg. Surg..

[89] Schwartze V.U., Jacobsen I.D. (2014). Mucormycoses Caused by *Lichtheimia* Species. Mycoses.

[90] Mohindra S., Gupta B., Gupta K., Bal A. (2014). Tracheal Mucormycosis Pneumonia: A Rare Clinical Presentation. Respir. Care.

[91] Lat A., Bhadelia N., Miko B., Furuya E.Y., Thompson G.R. (2010). Invasive Aspergillosis after Pandemic (H1N1) 2009. Emerg. Infect. Dis..

[92] Garcia-Vidal C., Barba P., Arnan M., Moreno A., Ruiz-Camps I., Gudiol C., Ayats J., Ortí G., Carratal J. (2011). Invasive Aspergillosis Complicating Pandemic Influenza A (H1N1) Infection in Severely Immunocompromised Patients. Clin. Infect. Dis..

[93] Ku Y.H., Chan K.S., Yang C.C., Tan C.K., Chuang Y.C., Yu W.L. (2017). Higher Mortality of Severe Influenza Patients with Probable Aspergillosis than Those with and without Other Coinfections. J. Formos. Med. Assoc..

[94] Robinson K.M. (2022). Mechanistic Basis of Super-Infection: Influenza-Associated Invasive Pulmonary Aspergillosis. J. Fungi.

[95] Song G., Liang G., Liu W. (2020). Fungal Co-Infections Associated with Global COVID-19 Pandemic: A Clinical and Diagnostic Perspective from China. Mycopathologia.

[96] Blaize M., Mayaux J., Nabet C., Nabet C., Lampros A., Marcelin A.G., Marcelin A.G., Thellier M., Thellier M., Piarroux R. (2020). Fatal Invasive Aspergillosis and Coronavirus Disease in an Immunocompetent Patient. Emerg. Infect. Dis..

[97] Alanio A., Dellière S., Fodil S., Bretagne S., Mégarbane B. (2020). Prevalence of Putative Invasive Pulmonary Aspergillosis in Critically Ill Patients with COVID-19. Lancet Respir. Med..

[98] Rafat Z., Ramandi A., Khaki P.A., Ansari S., Ghaderkhani S., Haidar H., Tajari F., Roostaei D., Ghazvini R.D., Hashemi S.J. (2022). Fungal and Bacterial Co-Infections of the Respiratory Tract among Patients with COVID-19 Hospitalized in Intensive Care Units. Gene Rep..

[99] Nayak N., Khan E., Panigrahi D. (2022). COVID-19 and Mucormycosis Coinfection: How Challenging It Is. Can. J. Infect. Dis. Med. Microbiol..

[100] Thabrew H., Sigera L.S.M., Anand A.P., Ramanayake R.A.D.M., Munasinghe M.V., Wickramasekara W.L.L.N., Jayasekera P.I. (2022). S5.5d SARS-CoV-2 Associated Invasive Fungal Sinus Infections; the Sri Lankan Perspective. Med. Mycol..

[101] Pasquier G., Bounhiol A., Robert Gangneux F., Zahar J.R., Gangneux J.P., Novara A., Bougnoux M.E., Dannaoui E. (2021). A Review of Significance of *Aspergillus* Detection in Airways of ICU COVID-19 Patients. Mycoses.

[102] Wang Y., Zhang L., Zhou L., Zhang M., Xu Y. (2022). Epidemiology, Drug Susceptibility, and Clinical Risk Factors in Patients with Invasive Aspergillosis. Front. Public Health.

[103] M B., T S., A U. (2023). Mucor Mycosis and Other Fungal Infections in COVID-19 Patients During Second Wave of Pandemic at a Tertiary Care Hospital. Asian J. Pharm. Clin. Res..

[104] Kumar D., Ahmad F., Kumar A., Bishnoi M., Grover A., Rewri P. (2023). Risk Factors, Clinical Manifestations, and Outcomes of COVID-19-Associated Mucormycosis and Other Opportunistic Fungal Infections. Cureus.

[105] Soltani S., Zandi M., Faramarzi S., Shahbahrami R., Vali M., Rezayat S.A., Pakzad R., Malekifar P., Pakzad I., Jahandoost N. (2022). Worldwide Prevalence of Fungal Coinfections among COVID-19 Patients: A Comprehensive Systematic Review and Meta-Analysis. Osong Public Health Res. Perspect..

[106] Pal R., Singh B., Bhadada S.K., Banerjee M., Bhogal R.S., Hage N., Kumar A. (2021). COVID-19-Associated Mucormycosis: An Updated Systematic Review of Literature. Mycoses.

[107] Fernández-García O., Guerrero-Torres L., Roman-Montes C.M., Rangel-Cordero A., Martínez-Gamboa A., Ponce-de-Leon A., Gonzalez-Lara M.F. (2021). Isolation of *Rhizopus microsporus* and *Lichtheimia* Corymbifera from Tracheal Aspirates of Two Immunocompetent Critically Ill Patients with COVID-19. Med. Mycol. Case Rep..

[108] Koukaki E., Rovina N., Tzannis K., Sotiropoulou Z., Loverdos K., Koutsoukou A., Dimopoulos G. (2022). Fungal Infections in the ICU during the COVID-19 Era: Descriptive and Comparative Analysis of 178 Patients. J. Fungi.

[109] Ezeokoli O.T., Gcilitshana O., Pohl C.H. (2021). Risk Factors for Fungal Co-Infections in Critically Ill COVID-19 Patients, with a Focus on Immunosuppressants. J. Fungi.

[110] Taghinejad Z., Asgharzadeh M., Asgharzadeh V., Kazemi A. (2021). Risk Factors for Mucormycosis in COVID-19 Patients. Jundishapur J. Microbiol..

[111] Crotta S., Davidson S., Mahlakoiv T., Desmet C.J., Buckwalter M.R., Albert M.L., Staeheli P., Wack A. (2013). Type I and Type III Interferons Drive Redundant Amplification Loops to Induce a Transcriptional Signature in Influenza-Infected Airway Epithelia. PLoS Pathog..

[112] Chang S.S., Zhang Z., Liu Y. (2012). RNA Interference Pathways in Fungi: Mechanisms and Functions. Annu. Rev. Microbiol..

[113] Salazar F., Brown G.D. (2018). Antifungal Innate Immunity: A Perspective from the Last 10 Years. J. Innate Immun..

[114] Rivera A., Lodge J., Xue C. (2022). Harnessing the Immune Response to Fungal Pathogens for Vaccine Development. Annu. Rev. Microbiol..

[115] Espinosa V., Dutta O., McElrath C., Du P., Chang Y.J., Cicciarelli B., Pitler A., Whitehead I., Obar J.J., Durbin J.E. (2017). Type III Interferon Is a Critical Regulator of Innate Antifungal Immunity. Sci. Immunol..

[116] Dutta O., Espinosa V., Wang K., Avina S., Rivera A. (2020). Dectin-1 Promotes Type I and III Interferon Expression to Support Optimal Antifungal Immunity in the Lung. Front. Cell. Infect. Microbiol..

[117] Kerr S.C., Fischer G.J., Sinha M., McCabe O., Palmer J.M., Choera T., Yun Lim F., Wimmerova M., Carrington S.D., Yuan S. (2016). FleA Expression in *Aspergillus fumigatus* Is Recognized by Fucosylated Structures on Mucins and Macrophages to Prevent Lung Infection. PLoS Pathog..

[118] Webb H.E., Brichta-Harhay D.M., Brashears M.M., Nightingale K.K., Arthur T.M., Bosilevac J.M., Kalchayanand N., Schmidt J.W., Wang R., Granier S.A. (2017). Salmonella in Peripheral Lymph Nodes of Healthy Cattle at Slaughter. Front. Microbiol..

[119] Lehrnbecher T., Schmidt S. (2019). Why are natural killer cells important for defense against Aspergillus?. Med. Mycol..

[120] Shallal A.F., Razaq C.H., Ahmed A.S., Ahmed S.A., Eyupoglu V., Salman R.S. (2026). Host Immune Responses to Pathogenic Fungi and Contemporary Therapeutic Strategies. J. Mycol. Truffle.

[121] Montaño D.E., Voigt K. (2020). Host Immune Defense upon Fungal Infections with Mucorales: Pathogen-Immune Cell Interactions as Drivers of Inflammatory Responses. J. Fungi.

[122] Zhang H., Zhu A. (2020). Emerging Invasive Fungal Infections: Clinical Features and Controversies in Diagnosis and Treatment Processes. Infect. Drug Resist..

[123] Wang H.C., Chang K., Lu P.L., Tsai K.B., Chen H.C. (2015). Fatal Invasive Aspergillosis: A Rare Co-Infection with an Unexpected Image Presentation in a Patient with Dengue Shock Syndrome. Clin. Respir. J..

[124] Bhatt K., Agolli A., H. Patel M., Garimella R., Devi M., Garcia E., Amin H., Domingue C., Del Castillo R.G., Sanchez-Gonzalez M. (2021). High Mortality Co-Infections of COVID-19 Patients: Mucormycosis and Other Fungal Infections. Discoveries.

[125] Peter Donnelly J., Chen S.C., Kauffman C.A., Steinbach W.J., Baddley J.W., Verweij P.E., Clancy C.J., Wingard J.R., Lockhart S.R., Groll A.H. (2020). Revision and Update of the Consensus Definitions of Invasive Fungal Disease from the European Organization for Research and Treatment of Cancer and the Mycoses Study Group Education and Research Consortium. Clin. Infect. Dis..

[126] Ruhnke M., Behre G., Buchheidt D., Christopeit M., Hamprecht A., Heinz W., Heussel C.P., Horger M., Kurzai O., Karthaus M. (2018). Diagnosis of Invasive Fungal Diseases in Haematology and Oncology: 2018 Update of the Recommendations of the Infectious Diseases Working Party of the German Society for Hematology and Medical Oncology (AGIHO). Mycoses.

[127] Ramage G., Kean R., Rautemaa-Richardson R., Williams C., Lopez-Ribot J.L. (2025). Fungal Biofilms in Human Health and Disease. Nat. Rev. Microbiol..

[128] Wang D., Zeng N., Li C., Li Z., Zhang N., Li B. (2024). Fungal biofilm formation and its regulatory mechanism. Heliyon.

[129] Naranjo-Ortiz M.A., Gabaldón T. (2019). Fungal Evolution: Major Ecological Adaptations and Evolutionary Transitions. Biol. Rev..

[130] Mannan M., Nabeela S., Mishra R., Uppuluri P. (2024). Host Immune Response against Fungal Biofilms. Curr. Opin. Microbiol..

[131] Zarnowski R., Noll A., Chevrette M.G., Sanchez H., Jones R., Anhalt H., Fossen J., Jaromin A., Currie C., Nett J.E. (2021). Coordination of Fungal Biofilm Development by Extracellular Vesicle Cargo. Nat. Commun..

[132] Von Borowski R.G., Trentin D.S. (2021). Biofilms and Coronavirus Reservoirs: A Perspective Review. Appl. Environ. Microbiol..

[133] He D., Fu C., Ning M., Hu X., Li S., Chen Y. (2022). Biofilms Possibly Harbor Occult SARS-CoV-2 May Explain Lung Cavity, Re-Positive and Long-Term Positive Results. Front. Cell. Infect. Microbiol..

[134] Peng J., Wang Q., Mei H., Zheng H., Liang G., She X., Liu W. (2021). Fungal Co-Infection in COVID-19 Patients. Aging.

[135] Mendonça A., Santos H., Franco-Duarte R., Sampaio P. (2022). Fungal Infections Diagnosis—Past, Present and Future. Res. Microbiol..

[136] Fraser M., Borman A.M., Thorn R., Lawrance L.M. (2020). Resistance to Echinocandin Antifungal Agents in the United Kingdom in Clinical Isolates of *Candida glabrata*: Fifteen Years of Interpretation and Assessment. Med. Mycol..

[137] Jung J., Park Y.S., Sung H., Song J.S., Lee S.-O., Choi S.-H., Kim Y.S., Woo J.H., Kim S.-H. (2015). Using Immunohistochemistry to Assess the Accuracy of Histomorphologic Diagnosis of Aspergillosis and Mucormycosis. Clin. Infect. Dis..

[138] Hammad M.A., Sulaiman S.A.S., Aziz N.A., Noor D.A.M. (2019). Prescribing Statins among Patients with Type 2 Diabetes: The Clinical Gap between the Guidelines and Practice. J. Res. Med. Sci..

[139] Yasmin F., Najeeb H., Naeem A., Dapke K., Phadke R., Asghar M.S., Shah S.M.I., De Berardis D., Ullah I. (2021). COVID-19 Associated Mucormycosis: A Systematic Review from Diagnostic Challenges to Management. Diseases.

[140] Soni S., Namdeo Pudake R., Jain U., Chauhan N. (2022). A Systematic Review on SARS-CoV-2-Associated Fungal Coinfections. J. Med. Virol..

[141] Dadwal S.S., Kontoyiannis D.P. (2018). Recent Advances in the Molecular Diagnosis of Mucormycosis. Expert Rev. Mol. Diagn..

[142] Martin-Souto L., Buldain I., Areitio M., Aparicio-Fernandez L., Antoran A., Bouchara J.P., Martin-Gomez M.T., Rementeria A., Hernando F.L., Ramirez-Garcia A. (2020). ELISA Test for the Serological Detection of Scedosporium/Lomentospora in Cystic Fibrosis Patients. Front. Cell. Infect. Microbiol..

[143] Guarner J., Brandt M.E. (2011). Histopathologic Diagnosis of Fungal Infections in the 21st Century. Clin. Microbiol. Rev..

[144] White S.K., Schmidt R.L., Walker B.S., E Hanson K. (2020). (1→3)-β-D-glucan Testing for the Detection of Invasive Fungal Infections in Immunocompromised or Critically Ill People. Cochrane Database Syst. Rev..

[145] Hashim Z., Neyaz Z., Marak R.S.K., Nath A., Nityanand S., Tripathy N.K. (2022). Practice Guidelines for the Diagnosis of COVID-19-Associated Pulmonary Aspergillosis in an Intensive Care Setting. J. Intensive Care Med..

[146] Fang W., Wu J., Cheng M., Zhu X., Du M., Chen C., Liao W., Zhi K., Pan W. (2023). Diagnosis of Invasive Fungal Infections: Challenges and Recent Developments. J. Biomed. Sci..

[147] Lehmann L.E., Hunfeld K.-P., Emrich T., Haberhausen G., Wissing H., Hoeft A., Stüber F. (2008). A Multiplex Real-Time PCR Assay for Rapid Detection and Differentiation of 25 Bacterial and Fungal Pathogens from Whole Blood Samples. Med. Microbiol. Immunol..

[148] Lahmer T., da Costa C.P., Held J., Rasch S., Ehmer U., Schmid R.M., Huber W. (2017). Usefulness of 1,3 Beta-d-Glucan Detection in Non-HIV Immunocompromised Mechanical Ventilated Critically Ill Patients with ARDS and Suspected *Pneumocystis jirovecii* Pneumonia. Mycopathologia.

[149] Tsang C.-C., Teng J.L.L., Lau S.K.P., Woo P.C.Y. (2021). Rapid Genomic Diagnosis of Fungal Infections in the Age of Next-Generation Sequencing. J. Fungi.

[150] Ibáñez-Martínez E., Ruiz-Gaitán A., Pemán-García J. (2017). Update on the Diagnosis of Invasive Fungal Infection. Rev. Esp. Quimioter..

[151] Cornely O.A., Alastruey-Izquierdo A., Arenz D., Chen S.C.A., Dannaoui E., Hochhegger B., Hoenigl M., Jensen H.E., Lagrou K., Lewis R.E. (2019). Global Guideline for the Diagnosis and Management of Mucormycosis: An Initiative of the European Confederation of Medical Mycology in Cooperation with the Mycoses Study Group Education and Research Consortium. Lancet Infect. Dis..

[152] Howard K.C., Dennis E.K., Watt D.S., Garneau-Tsodikova S. (2020). A Comprehensive Overview of the Medicinal Chemistry of Antifungal Drugs: Perspectives and Promise. Chem. Soc. Rev..

[153] Lin Y., Betts H., Keller S., Cariou K., Gasser G. (2021). Recent Developments of Metal-Based Compounds against Fungal Pathogens. Chem. Soc. Rev..

[154] Cavassin F.B., Baú-Carneiro J.L., Vilas-Boas R.R., Queiroz-Telles F. (2021). Sixty Years of Amphotericin B: An Overview of the Main Antifungal Agent Used to Treat Invasive Fungal Infections. Infect. Dis. Ther..

[155] Ellis D. (2002). Amphotericin B: Spectrum and Resistance. J. Antimicrob. Chemother..

[156] Pound M.W., Townsend M.L., Dimondi V., Wilson D., Drew R.H. (2011). Overview of Treatment Options for Invasive Fungal Infections. Med. Mycol..

[157] Melchers M., Van Zanten A.R.H., Heusinkveld M., Leeuwis J.W., Schellaars R., Lammers H.J.W., Kreemer F.J., Haas P.J., Verweij P.E., Van Bree S.H.W. (2022). Nebulized Amphotericin B in Mechanically Ventilated COVID-19 Patients to Prevent Invasive Pulmonary Aspergillosis: A Retrospective Cohort Study. Crit. Care Explor..

[158] Sheehan D.J., Hitchcock C.A., Sibley C.M. (1999). Current and Emerging Azole Antifungal Agents. Clin. Microbiol. Rev..

[159] Maertens J.A., Rahav G., Lee D.-G., Ponce-de-León A., Ramírez Sánchez I.C., Klimko N., Sonet A., Haider S., Diego Vélez J., Raad I. (2021). Posaconazole versus Voriconazole for Primary Treatment of Invasive Aspergillosis: A Phase 3, Randomised, Controlled, Non-Inferiority Trial. Lancet.

[160] Dannaoui E. (2017). Antifungal Resistance in Mucorales. Int. J. Antimicrob. Agents.

[161] Hong W., White P.L., Backx M., Gangneux J.P., Reizine F., Koehler P., Bentvelsen R.G., Cuestas M.L., Fakhim H., Jung J.I. (2022). CT Findings of COVID-19-Associated Pulmonary Aspergillosis: A Systematic Review and Individual Patient Data Analysis. Clin. Imaging.

[162] Baracaldo-Santamaría D., Cala-Garcia J.D., Medina-Rincón G.J., Rojas-Rodriguez L.C., Calderon-Ospina C.A. (2022). Therapeutic Drug Monitoring of Antifungal Agents in Critically Ill Patients: Is There a Need for Dose Optimisation?. Antibiotics.

[163] Denning D.W. (2003). Echinocandin Antifungal Drugs. Lancet.

[164] Rotta I., Sanchez A., Gonçalves P.R., Otuki M.F., Correr C.J. (2012). Efficacy and Safety of Topical Antifungals in the Treatment of Dermatomycosis: A Systematic Review. Br. J. Dermatol..

[165] Perfect J.R. (2017). The Antifungal Pipeline: A Reality Check. Nat. Rev. Drug Discov..

[166] Wu X., Zhang S., Li H., Shen L., Dong C., Sun Y., Chen H., Xu B., Zhuang W., Deighton M. (2020). Biofilm Formation of *Candida albicans* Facilitates Fungal Infiltration and Persister Cell Formation in Vaginal Candidiasis. Front. Microbiol..

[167] Rautemaa R., Ramage G. (2011). Oral Candidosis Clinical Challenges of a Biofilm Disease. Crit. Rev. Microbiol..

[168] Whaley S.G., Berkow E.L., Rybak J.M., Nishimoto A.T., Barker K.S., Rogers P.D. (2017). Azole Antifungal Resistance in *Candida albicans* and Emerging Non-Albicans *Candida* Species. Front. Microbiol..

[169] Kane A., Carter D.A. (2022). Augmenting Azoles with Drug Synergy to Expand the Antifungal Toolbox. Pharmaceuticals.

[170] Fiori B., Posteraro B., Torelli R., Tumbarello M., Perlin D.S., Fadda G., Sanguinetti M. (2011). In Vitro Activities of Anidulafungin and Other Antifungal Agents against Biofilms Formed by Clinical Isolates of Different *Candida* and *Aspergillus* Species. Antimicrob. Agents Chemother..

[171] Uppuluri P., Srinivasan A., Ramasubramanian A., Lopez-Ribot J.L. (2011). Effects of Fluconazole, Amphotericin B, and Caspofungin on *Candida albicans* Biofilms under Conditions of Flow and on Biofilm Dispersion. Antimicrob. Agents Chemother..

[172] Wagner C., Graninger W., Presterl E., Joukhadar C. (2006). The Echinocandins: Comparison of Their Pharmacokinetics, Pharmacodynamics and Clinical Applications. Pharmacology.

[173] Odds F.C., Brown A.J.P., Gow N.A.R. (2003). Antifungal Agents: Mechanisms of Action. Trends Microbiol..

[174] Spellberg B., Ibrahim A.S. (2010). Recent Advances in the Treatment of Mucormycosis. Curr. Infect. Dis. Rep..

[175] Ibrahim A.S., Spellberg B., Walsh T.J., Kontoyiannis D.P. (2012). Pathogenesis of Mucormycosis. Clin. Infect. Dis..

[176] Lei S., Chen X., Wu J., Duan X., Men K. (2022). Small Molecules in the Treatment of COVID-19. Signal Transduct. Target. Ther..

[177] Liu X., Li T., Wang D., Yang Y., Sun W., Liu J., Sun S. (2017). Synergistic Antifungal Effect of Fluconazole Combined with Licofelone against Resistant *Candida albicans*. Front. Microbiol..

[178] Nguyen T.L.A., Bhattacharya D. (2022). Antimicrobial Activity of Quercetin: An Approach to Its Mechanistic Principle. Molecules.

[179] Ribeiro F.D.E.C., Luzia E., Santos D.E.S., Fugisaki L.R.O., Oliveira L.D.D.E. (2020). Antifungal and Anti-Biofilm Effect of the Calcium Channel Blocker Verapamil on Non-Albicans *Candida* Species. An. Acad. Bras. Ciênc..

[180] Shekhar-Guturja T., Tebung W.A., Mount H., Liu N., Köhler J.R., Whiteway M., Cowen L.E. (2016). Beauvericin Potentiates Azole Activity via Inhibition of Multidrug Efflux, Blocks *Candida albicans* Morphogenesis, and Is Effluxed via Yor1 and Circuitry Controlled by Zcf29. Antimicrob. Agents Chemother..

[181] Li Y., Chang W., Zhang M., Li X., Jiao Y., Lou H. (2015). Diorcinol D Exerts Fungicidal Action against *Candida albicans* through Cytoplasm Membrane Destruction and ROS Accumulation. PLoS ONE.

[182] Maleki B., Sadeghian A.M., Ranjbar M. (2025). Impact of Vaccination against SARS-CoV-2 on Mortality Risk, ICU Admission Rate, and Hospitalization Length in Hospitalized COVID-19 Patients: A Cross-Sectional Study. BMC Infect. Dis..

[183] Mohammed I., Nauman A., Paul P., Ganesan S., Chen K.H., Jalil S.M.S., Jaouni S.H., Kawas H., Khan W.A., Vattoth A.L. (2022). The Efficacy and Effectiveness of the COVID-19 Vaccines in Reducing Infection, Severity, Hospitalization, and Mortality: A Systematic Review. Hum. Vaccin. Immunother..

[184] Ankamma Rao M., Jaradat E.K., Padma Devi M., Dhandapani P.B., Nalule R.M., Al-Hmoud M. (2025). Modeling COVID-19 Pneumonia and COVID-Associated Pulmonary Aspergillosis: Sensitivity Analysis and Optimal Control. BMC Infect. Dis..

[185] Lim H.X., Masomian M., Khalid K., Kumar A.U., MacAry P.A., Poh C.L. (2022). Identification of B-Cell Epitopes for Eliciting Neutralizing Antibodies against the SARS-CoV-2 Spike Protein through Bioinformatics and Monoclonal Antibody Targeting. Int. J. Mol. Sci..

[186] Chavda V.P., Prajapati R., Lathigara D., Nagar B., Kukadiya J., Redwan E.M., Uversky V.N., Kher M.N., Patel R. (2022). Therapeutic Monoclonal Antibodies for COVID-19 Management: An Update. Expert Opin. Biol. Ther..

[187] Cox M., Peacock T.P., Harvey W.T., Hughes J., Wright D.W., Willett B.J., Thomson E., Gupta R.K., Peacock S.J., Robertson D.L. (2023). SARS-CoV-2 Variant Evasion of Monoclonal Antibodies Based on in Vitro Studies. Nat. Rev. Microbiol..

[188] Wada D., Nakamori Y., Maruyama S., Shimazu H., Saito F., Yoshiya K., Kuwagata Y. (2022). Novel Treatment Combining Antiviral and Neutralizing Antibody-Based Therapies with Monitoring of Spike-Specific Antibody and Viral Load for Immunocompromised Patients with Persistent COVID-19 Infection. Exp. Hematol. Oncol..

[189] Filippatos C., Ntanasis-Stathopoulos I., Sekeri K., Ntanasis-Stathopoulos A., Gavriatopoulou M., Psaltopoulou T., Dounias G., Sergentanis T.N., Terpos E. (2023). Convalescent Plasma Therapy for COVID-19: A Systematic Review and Meta-Analysis on Randomized Controlled Trials. Viruses.

[190] Focosi D., Aiello T.F., Casadevall A., Cruciani M., D’Abramo A., De Marco P., Gachoud D., Garcia-Vidal C., Glingani C., Hueso T. (2026). Convalescent Plasma Restores Viral Clearance After Anti-Spike Monoclonal Antibody Failure in Immunocompromised COVID-19 Patients: A Systematic Review and Meta-Analysis of Individual Patient Data. Rev. Med. Virol..

[191] Bruch A., Kelani A.A., Blango M.G. (2022). RNA-Based Therapeutics to Treat Human Fungal Infections. Trends Microbiol..

[192] Makkar S.K. (2025). Advances in RNA-Based Therapeutics: Current Breakthroughs, Clinical Translation, and Future Perspectives. Front. Genet..

[193] Morio F., Lombardi L., Butler G. (2020). The CRISPR Toolbox in Medical Mycology: State of the Art and Perspectives. PLoS Pathog..

[194] Branda F., Petrosillo N., Ceccarelli G., Giovanetti M., De Vito A., Madeddu G., Scarpa F., Ciccozzi M. (2025). Antifungal Agents in the 21st Century: Advances, Challenges, and Future Perspectives. Infect. Dis. Rep..

